# Genome-wide association study and genotypic variation for the major tocopherol content in rice grain

**DOI:** 10.3389/fpls.2024.1426321

**Published:** 2024-10-08

**Authors:** Sara Kazemzadeh, Naser Farrokhi, Asadollah Ahmadikhah, Kourosh Tabar Heydar, Abdolali Gilani, Hossein Askari, Pär K. Ingvarsson

**Affiliations:** ^1^ Department of Cell and Molecular Biology, Faculty of Life Sciences & Biotechnology, Shahid Beheshti University, Tehran, Iran; ^2^ Chemistry and Chemical Engineering Research Center of Iran, Tehran, Iran; ^3^ Agricultural and Natural Resources Research Institute of Khuzestan, Ahwaz, Iran; ^4^ Department of Plant Biology, Swedish University of Agricultural Sciences, Uppsala, Sweden

**Keywords:** kinases, microProtein, quantitative trait loci (QTLs), transcription factor, transporter

## Abstract

Rice tocopherols, vitamin E compounds with antioxidant activity, play essential roles in human health. Even though the key genes involved in vitamin E biosynthetic pathways have been identified in plants, the genetic architecture of vitamin E content in rice grain remains unclear. A genome-wide association study (GWAS) on 179 genotypically diverse rice accessions with 34,323 SNP markers was conducted to detect QTLs that define total and α- tocopherol contents in rice grains. Total and α-tocopherol contents had a strong positive correlation and varied greatly across the accessions, ranging from 0.230-31.76 and 0.011-30.83 (μg/g), respectively. A total of 13 QTLs were identified, which were spread across five of the rice chromosomes. Among the 13 QTLs, 11 were considered major with phenotypic variation explained (PVE) greater than 10%. Twelve transcription factor (TF) genes, one microprotein (miP), and a transposon were found to be associated with the QTLs with putative roles in controlling tocopherol contents. Moreover, intracellular transport proteins, ABC transporters, nonaspanins, and SNARE, were identified as associated genes on chromosomes 1 and 8. In the vicinity of seven QTLs, protein kinases were identified as key signaling factors. Haplotype analysis revealed the QTLs *qAlph1.1, qTot1.1, qAlph2.1, qAlph6.1, qTot6.1*, and *qTot8.3* to have significant haplogroups. Quantitative RT-PCR validated the expression direction and magnitude of *WRKY39* (*Os02g0265200*), *PIP5Ks* (*Os08g0450800*), and *MADS59* (*Os06g0347700*) in defining the major tocopherol contents. This study provides insights for ongoing biofortification efforts to breed and/or engineer vitamin E and antioxidant levels in rice and other cereals.

## Introduction

**Figure 1:**
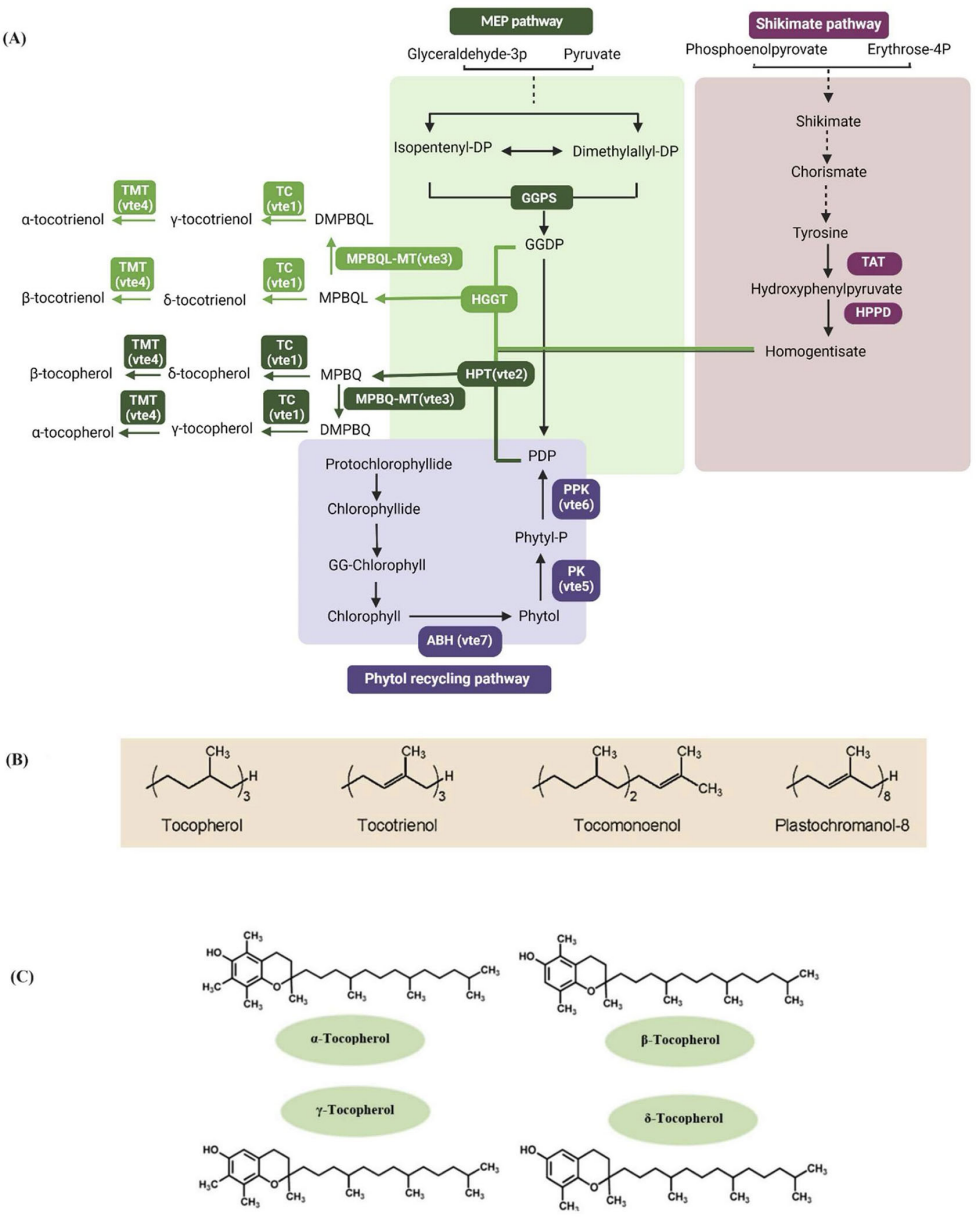
**(A)** Biosynthetic pathway of tococromonals-tocopherols and tocotrienols are formed from the combination of MEP (methylerythritol phosphate) and shikimate pathways. The shikimate pathway gives rise to a chromanol ring from HGA (homogentisate). MEP pathway provides the prenyl tail from *GGDP* (geranylgeranyl diphosphate) and *PDP* (phytyl diphosphate) for the synthesis of tocotrienol and tocopherol, respectively (Muñoz and Munne-Bosch, 2019). *VTE2* (a homogentisate phytyltransferase) condenses *PDP* and *HGA* to produce *MPBQ* (methylphytyl benzoquinol) (Sattler et al., 2004). In monocot lineage, HGA can also be condensed with *GGDP* by *HGGT1* (homogentisate geranylgeranyl transferase) to generate *MGGBQ* (methyl geranylgeranyl benzoquinol), the committed step for tocotrienol synthesis. *MPBQ* and *MGGBQ* are substrates for a series of methylations by *MPBQ*/*MGGBQ* methyltransferase (*VTE3*) and *γ-tocopherol methyltransferase* (*VTE4*) and cyclization by tocopherol cyclase (*VTE1*) whose sequence and numbers of reactions generate the α, β, δ, and γ isoforms of tocopherols and tocotrienols (Muñoz and Munne-Bosch, 2019). At the same time, *GGDP* for tocotrienol synthesis comes directly from the isoprenoid pathway. The generation of *PDP* for tocopherol synthesis is more complex and still not completely resolved. Differences in leaf tocopherol synthesis exist between monocots and dicots; tocopherol synthesized in seeds requires chlorophyll biosynthesis (Diepenbrock et al., 2017; Zhan et al., 2019), and the activity of *VTE7*, an alpha/beta hydrolase that interfaces with chlorophyll synthesis to release phytol (Albert et al., 2022). Phytol is then sequentially phosphorylated to *PDP* through the action of phytol kinase (*VTE5*) and phytol phosphate kinase (*VTE6*) (Valentin et al., 2006; Vom Dorp et al., 2015) **(B)** Tocopherol contains fully saturated aliphatic side chain, while the side chain of tocotrienol contains three extra trans double bonds. PC-8 has a similar unsaturated as tocotrienol but a longer side chain, whereas tocomonoenol has only one double bond on its side chain (Tian et al., 2007; Sadre et al., 2010; Stacey et al., 2016). **(C)** Tocopherol chemical structures feature a chromanol head and a prenyl tail. DMGGBQ, dimethylgeranylgeranylbenzoquinol; DMPBQ, dimethylphytylbenzoquinol; GGDR, geranylgeranyl diphosphate reductase; GGPS, geranylgeranyl diphosphate synthase; HPPD, hydroxyphenylpyruvate dioxygenase; HPT, homogentisate phytyl transferase; MPBQ-MT, MPBQ methyltransferase; TAT, tyrosine aminotransferase; TC, tocopherol cyclase; TMT, tocopherol methyltransferase; ABH, alpha/beta hydrolase; PK, Phytol kinase; PPK, Phytyl phosphate kinase. The figure was created at BioRender.com.

The nutritional quality of rice is important for the intake of essential nutrients, *esp*. in countries where rice is considered a staple (Wang et al., 2018). Rice grain contains a wide range of bioactive compounds such as vitamin E (mostly found in bran), γ-oryzanols, phenolic acids, flavonoids, and phytic acid (Shammugasamy et al., 2015). Vitamin E, also known as tocochromanol, an essential nutrient in humans, is being synthesized by some cyanobacteria and all plant tissues, including leaves and grains (Sattler et al., 2004; Mène-Saffrané et al., 2010). The vitamin E biosynthetic pathway (**Figure 1A**) is highly conserved in the plant kingdom (DellaPenna and Mène-Saffrané, 2011). Common features of all tocochromanols are a hydrophobic polyprenyl side chain with anchoring properties into bio-membranes and an antioxidant chromanol ring derived from homogentisate (Schuy et al., 2019). According to the various types of side chains, vitamin E can be defined as tocopherol (α-, β-, γ-, and δ), tocotrienol, plastochromanol-8 (PC-8), and tocomonoenol. All four tocochromanol isoprenoid side chains are being produced by GGDP synthases (Muñoz and Munne-Bosch, 2019; **Figures 1B, C**). Among the four types of tocopherols, α-tocopherol shows the highest antioxidant activity and is preferentially recognized by human cells (Grusak and DellaPenna, 1999; Havaux et al., 2005; Traber and Atkinson, 2007).

As a lipid-soluble antioxidant, vitamin E scavenges free radicals (Zingg, 2007). Vitamin E deficiency exacerbates the onset of many diseases, such as cancers, Alzheimer’s disease, and cardiovascular ailments (Sen et al., 2007; Falk and Munné-Bosch, 2010; Sozen et al., 2019). Vitamin E is essential in human diets and is also required for plant-environment adaptations (Niu et al., 2022). Vitamin E is most abundant in seeds, where they are essential for protecting membrane lipids, especially during seed desiccation, storage, and germination (Albert et al., 2022), effectively scavenging lipid peroxyl radicals, thus prolonging seed longevity (Lee et al., 2020). Elevated vitamin E levels in livestock feed results in meat with longer storage life, more desirable appearance, and greater consumer preference, presumably by inhibiting lipid peroxidation (Lipka et al., 2013). The probable role of vitamin E as a signaling molecule in plant cells has been recently reported (Ali et al., 2022).

Tocopherol biosynthesis has been most extensively investigated in *Arabidopsis thaliana*, where genes for the synthesis of homogentistic acid (*HGA*), phytyl diphosphate (*PDP*), and the core vitamin E pathway itself (*VTE1* through *VTE6*) were initially identified. GWAS of seed tocopherols in 814 Arabidopsis lines (part of the 1001 Arabidopsis Genome Panel, Alonso-Blanco et al., 2016) proposed the involvement of a novel seed-specific α/β hydrolase (*AtVTE7*). *AtVTE7* is targeted to the chloroplast envelope and accounts for 55% of the total seed tocopherols. Consistent with the results in Arabidopsis, the maize orthologous gene *ZmVTE7* controls 38% and 49% of total tocopherols in kernel and leaf, respectively (Albert et al., 2022). Of note is that *AtVTE7* was only detected in seeds with no control over the tocopherol content of the leaf. Several chromosomal loci, identified via genome-wide association studies (GWAS, have been reported to be associated with natural variation in the content and composition of vitamin E in different grains. For instance, Haddadi et al. (2012) identified *Vte4*, *HPPD*, *GST*, and *Droug1* to be related to tocopherol content in sunflower. Lipka et al. (2013) found an SNP (#591822) in maize grain to be significantly associated with *ZmVTE4* and *ZmVTE1*. Others have reported strong associations between *Vte4* and α-tocopherol content in maize grain (Lipka et al., 2013; Wang et al., 2018; Baseggio et al., 2019). Graebner et al. (2015) identified 13 SNPs associated with vitamin E biosynthesis in barley. In another study, two genes *homogentisate phytyltransferase* (*HPT*-*7H*) and *homogentisate geranylgeranyltransferase* (*HGGT*), were identified on barley chromosome 7H. These genes code for key enzymes controlling the accumulation of tocopherols in leaves and tocotrienols in grains, respectively (Schuy et al., 2019; **Figure 1A**). Wang et al. (2015) performed GWAS using 1.44 million high-quality SNPs acquired from the re-sequencing of 137 accessions of a diverse rice core collection. This led to the identification of 13 candidate genes, of which *γ-tocopherol methyltransferase* (*OsγTMT*) was identified as the major gene controlling α-tocopherol content.

Although much information about tocopherol and biosynthesizing genes has accumulated, the knowledge concerning the molecular mechanisms of transport, turnover, signaling, and regulation of tocopherol biosynthesis remains fragmentary. Despite the impact of some transcription factors (TFs) in the methylerythritol phosphate (MEP) and shikimate (SK) pathways (**Figure 1A**), the regulatory mechanism in the tocopherol pathway remains unclear. Synthetic zinc finger transcription factors (ZFP-TFs) were designated to upregulate the expression of the endogenous Arabidopsis *γ-tocopherol methyltransferase* gene. This gene encodes the enzyme responsible for the conversion of γ-tocopherol to α-tocopherol. Overexpression of ZFP-TF under the control of the seed-specific promoter caused a 20-fold increase in α-tocopherol compared to control in Arabidopsis seeds (Van Eenennaam et al., 2004). In tomato, the overexpression of a MYB, *ODORANT1*, led to a significant increase in phenylpropanoid content concomitantly with transcriptional upregulation of several SK pathway genes (Dal Cin et al., 2011). Downregulation of *DET1*, which is involved in suppressing light responses in the absence of light in tomato fruit, resulted in increased contents of carotenoids, flavonoids, and tocopherol (Enfissi et al., 2010).

In addition to TFs, microproteins (miPs) are important regulatory factors consisting of small (5-20 kilodaltons) proteins known to regulate gene expression at the post-translational level through protein-protein interactions (Straub and Wenkel, 2017). The first miP family reported in plants was the LITTLE ZIPPER (ZPR) family of proteins (ZPR1-4). ZPR has a leucine zipper domain that interacts and post-translationally regulates other leucine zipper-containing proteins to modulate plant development (Wenkel et al., 2007). As newly discovered genes, very little is currently known about the structure and function of microproteins, making these genes interesting targets for discovering new biological mechanisms (Rathore et al., 2018). Transposable elements (TEs) are other genomic elements that have also been described as controlling elements, due to their ability to influence gene expression reversibly (Pachamuthu and Borges, 2023). Only a few TEs with regulatory functions have been discovered in rice to date, and their activities are mostly induced by artificial treatments (*e.g*., tissue culture and hybridization). *Tos17*, belonging to the Copia superfamily, is the first active retrotransposon isolated from rice (Hirochika et al., 1996), which exhibits a high level of transposition activity under tissue culture and has been used to induce rice mutants for functional genomics studies (Hirochika, 2001). Although tocopherols have been mostly related to photosynthetic tissues, the accumulation of tocopherols occurs in several non-photosynthetic tissues. The recent discovery of an α-tocopherol-binding protein (TBP, Muñoz and Munne-Bosch, 2019) has altered our view on tocopherol transport in plants, showing intraorganellar vitamin E transport within chloroplasts. TBPs may facilitate the rapid exchange of tocopherols between intraorganellar membranes in chloroplasts (Muñoz and Munne-Bosch, 2019).

Here, we investigated the regulatory genes (TFs, miPs, and transposons) and genes involved in the transport and signaling of tocopherol biosynthesis using a natural rice population. Rice is a model plant with great resources of genetic information. Collectively, our findings provide genetic information for increasing/modulating tocopherol content in rice, other cereals and crops through molecular breeding strategies. Moreover, the candidate genes in this study can be used in the metabolic engineering of microorganisms for the industrial production of tocopherols which currently are synthesized chemically for pharma.

## Materials and methods

### Plant material

A collection of 282 accessions of rice (*Oryza sativa*) from 82 countries was obtained from the International Rice Research Institute (IRRI), Philippines (**Supplementary Table S1**), in addition to three local cultivars included as check varieties (Sadri, Sang Tarom, Dom Siah Kalat). The experiment was conducted at the Agricultural Research Station, Shavoor, Ahwaz, Iran (48°27’ E, 31°50’ N) during the 2021-2022 cultivation season. The genotypes were planted in an augmented block design (Federer and Raghavarao, 1975) with three check varieties in every block (3 replicates). The land was fertilized using superphosphate triple (150 kg/ha) and potash (150 kg/ha) at the plowing stage. All seeds were harvested at maturity and stored in dry conditions at 4°C. A total of 179 accessions, belonging to *indica* (42), japonica (79), aus (21), aromatic (4), and admix (33), in addition to local cultivars, completed the growing season and used in our subsequent studies.

### SNP genotyping data

The development and sequencing of an SNP hybridization array for the rice population have previously been described by Zhao et al. (2011). Data for the rice 44.1 K SNPs array was downloaded from the Gramene portal (http://gramene.org) for all the rice accessions included in the study. SNP loci were further filtered to produce a high-quality set with minimum allele frequencies (MAF) greater than 0.05 in TASSEL v5.0 (Bradbury et al., 2007). After excluding low-quality and monomorphic loci, 34,323 SNP loci were retained.

### Analysis of rice grain tocopherol content

Rice grains (2 g) were crushed for 1 min and vortex-mixed in *n*-hexane (3:1 solvent: powder) for 1 min (Hu et al., 1996). The mixture was wrapped in aluminium foil to reduce light exposure and shaken in a rotary shaker at 220 rpm, 26°C for 16 h. The next day, samples were centrifuged at 8000 ×g for 10 min at 4°C. Supernatant was poured into a fresh tube and placed at 22°C for solvent evaporation. The remaining oil (500 µl) was passed through a PTFE filter (0.45 µm) and mixed with acetonitrile in a ratio of 1:1.

Tocopherol contents were determined by high-performance liquid chromatography (LC pump K-1001, KNAUER, Germany) coupled with a fluorescence detector (Shimadzu RF - 10Axl, Kyoto, Japan; excitation of 295 nm and an emission of 330 nm). The mobile phase was 100% acetonitrile at a flow rate of 1 ml.min^-1^ at a column temperature of 40°C. The oil injection volume was 20 ml. A C18 reverse phase column (GALAK, 150 mm length, 5 µm particle sizes, 100 A˚, 330 m^2^. g^-1^) was used to identify tocopherols. Amounts of tocopherol were calculated based on the established standard curve under the same HPLC conditions.

### GC- MS analysis

The fatty acid profile of grains of selected rice accessions was determined using a Gas Chromatogram (Agilent 6890 series, Palo Alto, CA, USA) attached to a mass spectrometer (Agilent 5973 Network mass selective detector, Delaware, USA). The FAMEs (*fatty acid methyl esters)* of the oils were separated using a CP-SIL 8CB-MS FS 50 × 2.25 column. The column oven temperature was initially maintained at 60°C for 5 min, increased at 5°C.min^-1^ to 280°C, and kept at 280°C for 15 min. The carrier gas was helium with a flow rate of 1.5 ml.min^-1^. Rice grain oil (0.5 ml) was dissolved in 1 ml hexane, reaching 5 ml with 2 M methanolic KOH. The mixture was stirred for 1 h at 22°C and centrifuged at 4,000 rpm for 10 min. The upper layer of *n*-hexane containing FAME was pipetted from the solution, placed in a 2 ml glass vial, and stored below -4°C until GC-MS. The composition was reported as a relative percentage of the total peak area. The FAMEs and essential oil constituents were identified by matching their mass spectra with the peaks in the library (NIST) database (Hagr and Adam, 2020).

### Population structure analysis

All high-quality SNPs were sampled to calculate population structure (Q) and kinship (K). Q and K matrices were used as covariates in the models for trait-marker association analysis. Principal component analysis (PCA) was conducted to summarize the genetic structure and variation present in the collection. The analyses to obtain principal components matrices of the accessions were performed using rMVP in R (Yin et al., 2021). Kinship analysis was also performed using 34,323 enumerated SNP data markers from 179 genotypes. The input file was prepared using TASSEL v.5.0, and the kinship matrix was obtained using the Popkin package in R. A heat map of kinship relationships was generated using the gplots package in R.

The normality of frequency distribution for the phenotypic data was tested using skewness and kurtosis tests in R. The ANOVA was carried out using the Augmented Block Design using R agricolae package 1.4.0. The broad-sense heritability (*H^2^b*) and narrow-sense heritability (*H^2^n*) of the alpha and total tocopherol were estimated using the heritability package in R (https://cran.r-project.org/web/packages/heritability/index.html) by the following equation: *H^2^b=V_G_/V_P_*, *H^2^n=V_A_/V_P_* where V_G_ is the genotypic variance, V_P_ is the phenotypic variance, and V_A_ is the additive variance.

### Linkage disequilibrium estimation

The filtered SNP data was used to calculate linkage disequilibrium (LD) between SNPs using r^2^ in a sliding window of 50 markers using TASSEL v5.0 (Bradbury et al., 2007). Graphs depicting the decay of LD with the physical distance between SNPs were created by aod, dplyr, and stringr and visualized using ggplot2 in R. The distance across the chromosome where r^2^ dropped to half its maximum value was called the LD decay distance (Huang et al., 2010).

### GWAS analysis

Genotypic data such as single nucleotide polymorphisms (SNPs), phenotypic data, and covariates (Q and K matrices) were collected to perform GWAS using rMVP (Yin et al., 2021). To ensure accuracy and to minimize the false-positive rate, two models were utilized independently: i) Mixed linear model (MLM; Price et al., 2006) and ii) Fixed and Random Model Circulating Probability Unification (FarmCPU; Liu et al., 2016). MLMs are models that account for both fixed and random effects in a dataset, while FarmCPU is a method that employs MLMs to identify genetic associations between traits and multiple markers in genomic data. This model uses a compressed MLM to improve computational efficiency and allows for the analysis of large datasets. Manhattan plots with suggested threshold lines were produced using rMVP, considering the very low LD decay distance in rice (Mather et al., 2007). The QTL span was defined as the location of significantly associated SNPs ± LD decay distance. Significant QTLs with –log10(*p*) ≥ 4 for αT and total tocopherol contents were determined based on Q-Q plot (Galwey, 2023). To reduce QTL redundancy, if overlaps were observed between two or more QTL flanking areas, they were combined into one QTL (Chen et al., 2014; Wang et al., 2015; Guo et al., 2018). Genes annotated as either involved in vitamin E biosynthesis or the closest gene near each QTL peak were proposed to be the most likely candidate genes.

### Bioinformatics and gene identification

To identify genes underlying the QTLs of vitamin E content that overlapped with the genomic regions (*i.e*., their associated SNPs), the genes were checked at IRGSP-1.0 (https://rapdb.dna.affrc.go.jp/). The flanking region of each SNP marker was chosen based on the distance of LD decay (Mather et al., 2007), and checked for map order uncertainty and LD. To ascertain whether candidate genes underlying the detected QTLs, which were previously cloned and functionally annotated, a thorough literature search was done for each gene. The online Plantpan3 tool (http://plantpan.itps.ncku.edu.tw/, Chen et al., 2019) was used to check each corresponding promoter and co-expression analysis of the main genes (Mène-Saffrané, 2017) in tocopherol biosynthesis pathway. The STRING tool (Search Tool for the Retrieval of Interacting Genes - https://string-db.org/) was employed to construct the protein association networks between candidate gene and main genes involved in tocopherol biosynthesis, with a confidence parameter set to a threshold of 0.40 and a maximum number of interactors in the first and second shells. The Cytoscape v3.9.1 software was used for network visualization. The miPFinder algorithm was used to identify the microproteins among the genes near detected QTLs. The effect of each associated SNP was annotated using SnpEff: rice genome annotation information from the RAP database (RAP-DB, https://rapdb.dna.affrc.go.jp/) was used in the SnpEff analysis. MapMan software (ver. 3.6.0RC1) was used to map transcriptomic data. Osa_RAPDB_mapping files were downloaded from the Map-Man store server (http://mapman.gabipd.org/web/guest/mapmanstore).

### Haplotyping

Haplotype analyses were performed, and the outputs were visualized using geneHapR (Zhang et al., 2023). The phenotypic value of each haplotype was assessed by calculating the average phenotypic value across the germplasm with each type of SNP locus linked to a specific target trait. In this study, favorable haplogroups were defined as the haplotype that showed the highest average values over other haplotypes. Haplotypes with heterozygous or missing data were removed.

### Epistatic interaction (QQ) analysis

Epistatic interactions between significant QTLs obtained via GWAS (−Log_10_ (*p*) > 4.0 in FarmCPU model) for total and α-tocopherol were analyzed. For QQ analysis, the snpPairInteraction function in the FRGEpistasis of R package (Zhang et al., 2014) was used and determined to be significant at a *P*-value ≤ 0.05. The Circlize package was used to visualize the significant epistatic interactions (Gu et al., 2014).

### Tissue-specific expression of candidate genes

The expression profiles of the candidate genes in various tissues were obtained from the Rice Genome Annotation Project database (RGAP, http://rice.plantbiology.msu.edu/), including shoots (library name in NCBI SRA: SRR042529), leaves-20 days (SRX100741), seed-5 DAP (days after pollination; SRX100749) and seed-10 DAP (SRX100755). A heatmap was generated to visualize the gene expression patterns across the different tissues. Functional enrichment analyses of candidate genes were depicted using MapMan 3.0.0 software based on the functional annotated file osa_RAPDB_mapping (https://MapMan.gabipd.org/MapManstore).

### Quantitative reverse transcription polymerase chain reaction

Two selected genotypes, #g95 (low tocopherol) and #g217 (high tocopherol), were grown in the spring of 2023, and fertilized three times with complete fertilizer. The genotypes were sampled at milky (7 days post pollination (DPP)), doughy (14 DPP), and mature developing seed (28 DPP) stages. Sampled seeds were wrapped in foil and placed in liquid nitrogen. Total RNA was isolated from seeds in different developmental stages using GenUP™ Total RNA Mini Kit (biotechrabbit GmbH, Berlin, Germany). First-strand cDNA was synthesized from total RNA using Thermo Scientific RevertAid RT Kit (#K1691, Thermo Fisher Scientific). qRT-PCR was performed using SYBR Green (FP205, Tiangen) reagents on a Real-Time PCR machine (T100 Thermal Cycler, Bio-Rad). All qRT-PCRs were performed in three independent replicates, and the relative expression levels were calculated using the 2^–ΔΔCt^ method (Livak and Schmittgen, 2001). Statistical significance of Ct differences between low (#g95) and high (#g217) tocopherol genotypes was calculated with a two-way ANOVA, employing Tukey’s post-test comparisons, using the GraphPad Prism 9.5.1 software.

The *OsActin* (*Os03g0718100*) was used as an internal control for expression normalization. Primer pairs used for the rice cDNA amplification were designed using Primer3Plus (https://www.primer3plus.com, **Supplementary Table S8**).

### Gene ontology enrichment and KEGG pathway determination

ShinyGO v0.75 (http://bioinformatics.sdstate.edu/go/) served as a web-based tool for exploring GO term enrichment and KEGG analysis for candidate genes (Ge et al., 2020). The search was conducted against the *Oryza sativa japonica* group.

## Results

### Phenotypic variation

In general, alpha and total tocopherol phenotypes were skewed towards lower values for the panel of 179 rice accessions (**Supplementary Figure S1**). Alpha-tocopherol content was significantly correlated with total tocopherol content (*r^2^* = 0.97). The correlation was consistent with the fact that alpha is the major tocopherol in rice grain and contributes greatly to the total tocopherol content in rice (**Supplementary Table S2**). The total tocopherol content in the accessions varied widely from 0.230 to 31.76 (µg/g), with an average value of 3.70 (µg/g) and a coefficient of variation (C.V.) of 6.7%. alpha-tocopherol contents in the seeds of the 179 accessions varied from 0.011 to 30.83 (µg/g), with a mean value of 3.30 (µg/g) and a C.V of 7.3%. The alpha/total tocopherol ratio of accessions in each rice subpopulation was calculated. The *indica* genotypes had a higher alpha/total tocopherol ratio than other accessions. Among the 179 rice accessions, the top five accessions with the highest alpha and total tocopherol contents were g253, g252, g120, g217, and g134. Estimates of *H^2^b* and *H^2^n* were 0.86 and 0.76 for alpha-tocopherol and 0.87 and 0.79 for total tocopherol content, respectively. Estimated genotypic variance (V_G_) and additive variance (V_A_) accounted for the alpha-tocopherol were 29.75 and 26.64, and for total tocopherol were 30.71 and 27.88, respectively (**Table 1**). Relatively high heritability values suggest that phenotypic-based selection for tocopherol content can be used effectively. These results showed that tocopherol content is mainly influenced by genetic factors with limited effect from environmental factors (Yu et al., 2022). Therefore, the tocopherol content of rice in this study was appropriate for GWAS.

**Table 1:** Statistical and variation analysis of tocopherol (toc) content in the tested rice population (n = 179).

Traits	Mean (µg/g)	Range (µg/g)	C.V%	H^2b^	H^2n^	VA	VG
**Total toc content**	3.70	0.230 - 31.76	6.7	0.87	0.79	27.88	30.71
**α-Toc content**	3.30	0.011 - 30.83	7.3	0.86	0.76	26.64	29.75

### Population stratification

**Figure 2:**
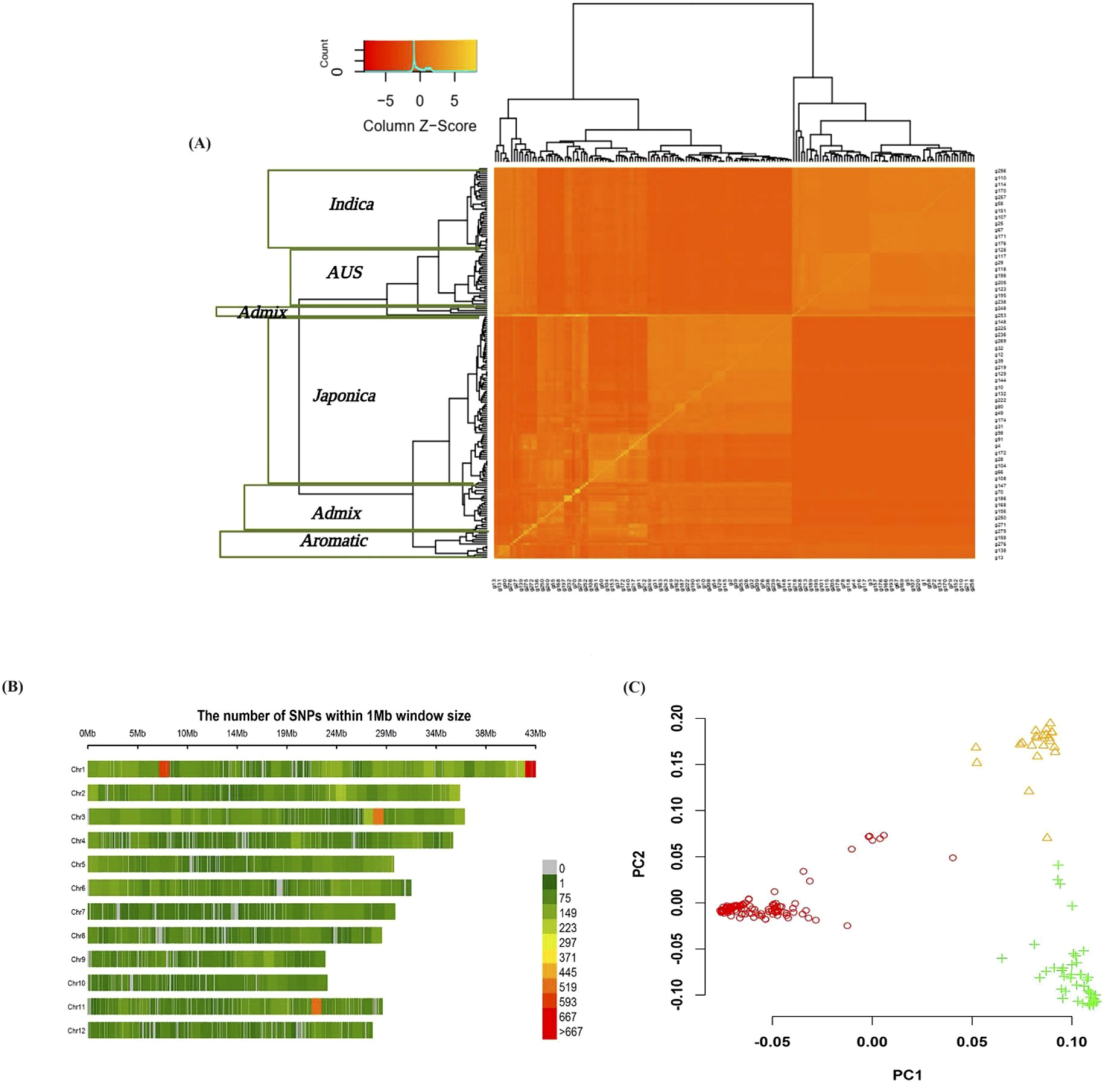
**(A)** Van Raden kinship matrix - heat map of the marker-based kinship (K) matrix for 179 rice accessions. Matrix calculated using 34,323 SNPs. **(B)** The chromosomal distribution of SNPs used in 1 Mbp window. The number of markers on each chromosome varies, with chromosome 9 having the least markers and chromosome 1 having the highest. **(C)** PCA plot used to analyze the population structure showing three major subpopulations (shown in different colors) in the current panel.

Rice accessions in our panel have previously been classified into five subpopulations: *Japonica*, *Indica*, *AUS*, *Aromatic*, and *Admix*, according to (Zhao et al., 2011). The diversity level and stratification of the accessions were examined before performing GWAS. A kinship matrix was used to summarize the distribution of pairwise relative relationship coefficients among the accessions in the association panel based on SNP information. A phylogenetic tree and heat map of the values in the kinship matrix were constructed from the SNPs, both of which showed relatedness among the accessions (**Figure 2A**).

### Markers statistics, LD decay, and GWAS

**Figure 3:**
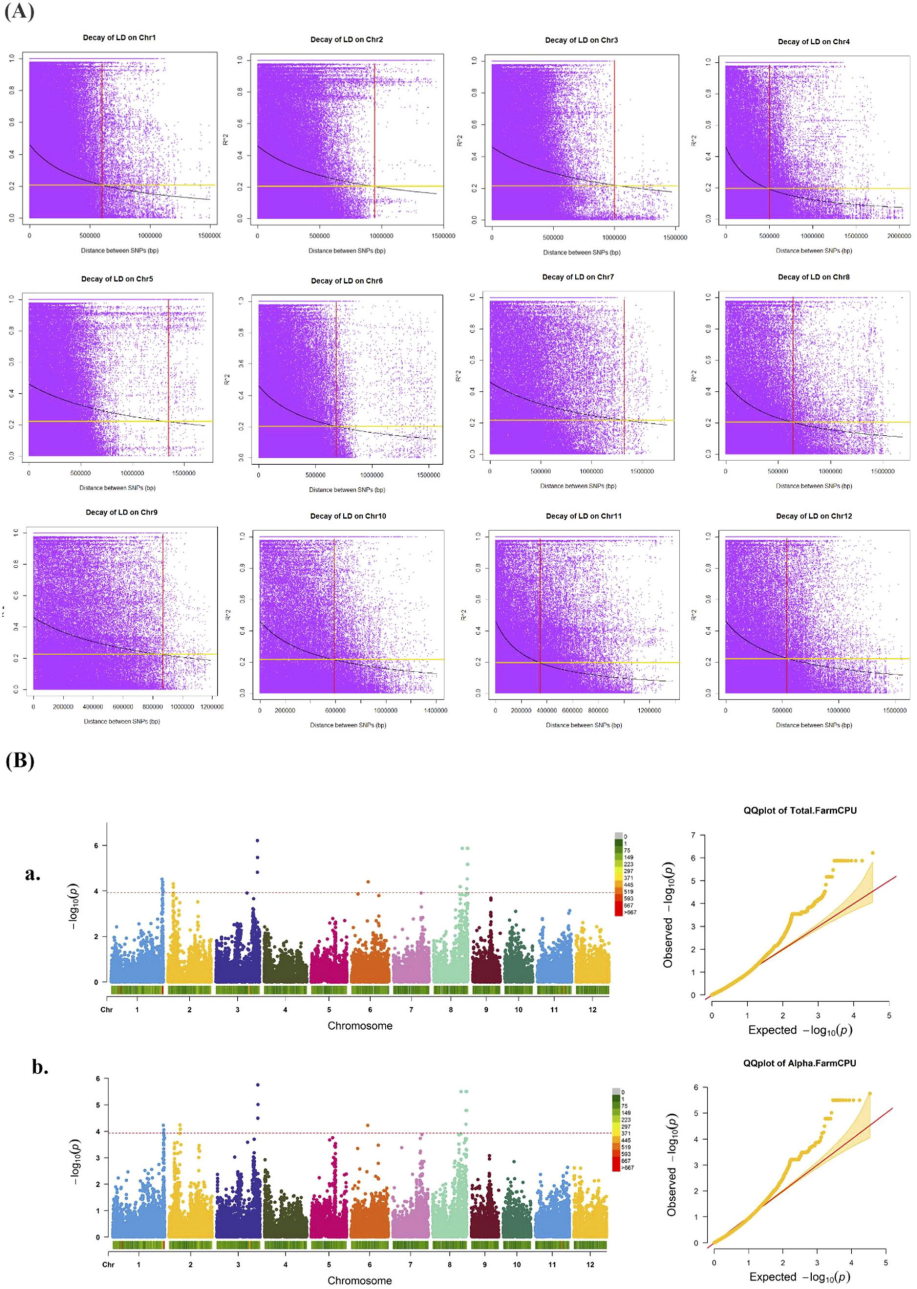
**(A)** The decay of LD along physical distances on 12 rice chromosomes was computed using SNP data of 179 rice accessions. A scatter *r* ^2^ against physical distance showed a clean pattern of LD decay in the 179 rice accessions. A critical value of the determination coefficients *r* ^2^ > 0.2 was determined to be the threshold for LD decay. **(B)** Manhattan and Q-Q plots. (a) Total tocopherol, (b) α-tocopherol. The FarmCPU model resulted in more associations than the MLM model, which revealed 13 significant QTLs with –log_10_(*p*) ≥ 4 for αT and total tocopherol contents.

A total of 34,323 SNPs distributed across 12 chromosomes were examined. The number of markers on each chromosome varied, with chromosome 9 having the fewest markers and chromosome 1 having the largest number (**Figure 2B**). According to the PCA plot (**Figure 2C**), three major subpopulations were evident in the current association panel. The LD dropped to half of its maximum value at a distance of 300-1300 kb across the 12 chromosomes (**Figure 3A**). Multi-locus association analyses were carried out using MLM and FarmCPU models (**Supplementary Figure S2**). Quantile-quantile (Q-Q) plots suggested that the FarmCPU model better fits the expected distribution. Accordingly, FarmCPU was used for all subsequent analyses to generate the corresponding Manhattan plots for alpha and total tocopherols (**Figure 3B**). A total of 74 (αT: 27, total tocopherol: 47) SNPs were significantly associated with tocopherol contents. These significant SNPs were located on chromosomes 1, 2, 3, 6, and 8 (**Supplementary Table S3**). Thirteen significant QTLs with –log_10_(*p*) ≥ 4 for the contents of alpha and total tocopherols were detected. The strongest QTL for αT and total tocopherol was located on chromosome 3 (**Supplementary Table S3**, **Figure 3B**–a, b). The flanking regions (SNP position ± LD decay) of the 13 QTLs were checked for the presence of the marker-associated genes. For each QTL, the Phenotypic Variation Explained (PVE) was calculated. The PVE for all QTLs with –log10(*p*) ≥ 4 was greater than 10%, except for the QTLs on chromosome 2 (*qTol2.1*, *qAlph2.1*). Therefore, of the 13 QTLs, 11 were considered major and two (*qTol2.1*, *qAlph2.1*) were minor (**Supplementary Table S3**). Various regulatory (TFs, miPs, and transposons) and functional genes with roles in cellular transport and signaling were found near the mapped QTLs. Among these genes, 51 primary TFs (αT = 22 and total tocopherol = 29) were identified using PlantTFDB5.0 (http://planttfdb.gao-lab.org/; **Supplementary Table S4**). A microprotein (miP; RGP-2) on chromosome 1 and a transposon (*Os03g0822100*) on chromosome 3 were also associated with tocopherol content. *RGP-2* with a length of 116 amino acids, was for the first time identified in our study. Transporter genes (9) from three families of ABC transporters, nonaspanins, and SNARE proteins on chromosomes 1 and 8 were also associated with tocopherol content (**Table 2**). Protein kinases were identified to be associated with seven QTLs as the probable key signaling factors and belonging to the receptor-like kinases (RLKs), S-domain subfamily of receptor-like kinases (SDRLKs), calcium-dependent protein kinase (CDPK), NAD kinase, cyclin-dependent kinase (CDK), nucleoside monophosphate (NMP) kinase and lipid kinase family (**Table 2**).

**Table 2:** Candidate genes identified via GWAS involved in vitamin E biosynthetic pathway.

Gene	SNP	Gene name	QTL name	Distance (bp)	LOP	Functional annotation	Reference
*Os08g0554900*	id8007643	*Nonaspanin (TM9SF)*	*qAlph8.2, qTot8.2*	88,339	5.9	transmembrane 9 superfamily	
*Os08g0555200*	id8007643	*Nonaspanin (TM9SF)*		116,667	5.9		
*Os08g0555300*	id8007643	*Nonaspanin (TM9SF)*		119,816	5.9		
*Os08g0558600*	id8007643	*VAMP727*		251,888	5.9	knockdown of the *Os08g0558600* gene could lead to a reduced tiller number phenotype with no heading	Wang et al., 2013
*Os08g0564100*	id8007982	*ABCF4*		122,842	5.2	ABC transporter f family member 4	
*Os08g0564300*	id8007982	*ABCB22*		106,884	5.2	The ABCB proteins can serve as auxin influx/efflux transporter and transporters for secondary metabolite as well as iron	Xu et al., 2014
*Os08g0563300*	id8007982	*BET11*		165,132	5.2	a critical role in the membrane fusion step of the vesicular transport system	Bao et al., 2012
*Os01g0966100*	dd1002535	*ABCD2*	*qAlph1, qTot1.1*	8,776	4.3	ABC transporter superfamily ABCD subgroup member 2	
*Os08g0544400*	id8007450	*ABCG45*	*qTot8.2*	67,773	4.1	high expression in reproductive stages as well as in the stress- and hormone- (IAA and BAP treatments) treated samples	Gupta et al., 2019
*Os03g0821900*	id3016979	*RLCK118*	*qAlph3.1, qTot3.1*	95,128	6.2	positively regulate chitin- and PGN- induced responses in rice/ positive regulators of rice immunity and plant development	Li et al., 2017; Fan et al., 2018; Xiao et al., 2022
*Os03g0825300*	id3016979	*RLCK119*		65,355	6.2	Serine/threonine protein kinase domain containing protein.	
*Os03g0825800*	id3016979	*RLCK120*		87,808	6.2	Protein kinase	
*Os03g0828800*	id3016979	*SDRLK60*		210,372	6.2	S-Domain receptor like kinase-60	
*Os08g0540400*	id8007643	*CDPK21*	*qTot8.2*	150,432	5.9	involved in multiple stress signaling pathways	Mittal et al., 2017
*Os01g0957000*	id1027324	*NADK1*	*qAlph1.1, qTot1.1*	31,361	4.5	NAD kinase, Mediation of intracellular redox balance, Drought tolerance	
*Os01g0957100*	id1027324	*Protein kinase*		35,288	4.5	–	
*Os01g0958000*	id1027324	*CDKC1*		80,262	4.5	key regulatory roles in diverse cellular functions, including cell-cycle progression, transcription and translation	Kitsios et al., 2008
*Os01g0960400*	id1027324	*Protein kinase*		196,309	4.5	–	
*Os01g0965400*	dd1002410	*YGL8*		43,018	4.4	chlorophyll biosynthesis gene	Peng et al., 2019
*Os01g0964400*	dd1002410	*RLCK56- receptor-like kinases (RLKs)*		76,058	4.4	Receptor-like Cytoplasmic Kinase 56	
*Os02g0182600*	id2002458	*Similar to Protein kinase*	*qTol2.1*	58,432	4.3	–	
*Os02g0186500*	id2002458	*RLCK64*		154,668	4.3	Receptor-like Cytoplasmic Kinase 64	
*Os08g0453800*	id8006337	*CDKC*	*qTot8.3*	141,978	4.2	involved in developmental events as well as in the plant salt stress response mechanism through an ABA-signaling pathway	Huang et al., 2008
*Os08g0446200*	id8006210	*PEPR1- receptor kinase*		42,933	4.2	defense signaling	Shinya et al., 2018
*Os08g0446301*	id8006210	*INRPK1c/ST-Pks*		37,438	4.2	–	
*Os08g0450800*	id8006210	*PIP5K- lipid kinase*		206,815	4.2	phosphatidylinositol signaling pathway and essential functions in growth, development, and biotic and abiotic stresses responses	Zhang et al., 2020
*Os03g0822100*	id3016979	*Similar to Transposase*	*qAlph3.1, qTot3.1*	76,895	6.2	Similar to Transposase	
*Os01g0974701*	ud1001752	*RGP-2*	*qTot1.1*	21,837	4	Similar to RNA-binding glycine rich protein	
*Os03g0820400*	id3016979	*ZFP15*	*qAlph3.1, qTot3.1*	174,970	6.2	Improvements in salinity and drought tolerance	Zhang et al., 2012; Wang et al., 2022
*Os03g0820300*	id3016979	*ZFP182*		178,494	6.2	salt tolerance related genes	Ning et al., 2018
*Os03g0818700*	id3016979	*MYB-like*		252,070	6.2	Similar to MYB transcription factor	
*Os03g0818800*	id3016979	*EREBP33*		244,481	6.2	tolerance to cold stress, drought response	Yang et al., 2023; Maghraby and Alzalaty, 2024
*Os08g0543900*	id8007643	*bZIP68*	*qTot8.2*	31,372	5.9	salt responsive; positively regulates ABA-independent osmotic stress signaling	Hossain et al., 2016; Zhou et al., 2022
*Os08g0477900*	id8006747	*bHLH042*	*qAlph8.1, qTot8.1*	50,709	5.9	basic helix-loop-helix protein 042	
*Os08g0549600*	id8007643	*OsbZIP69*		99,349	5.9	promotes flowering in rice	Cerise et al., 2021
*Os06g0344900*	id6008406	*NAC92*	*qAlph6.1, qTot6.1*	201,092	4.4	role in drought stress by regulating the related genes involved in drought tolerance	Wang et al., 2022
*Os06g0347700*	id6008406	*OsMADS59*		8,696	4.4	Phenylpropanoid biosynthesis pathway	Sun et al., 2023
*Os06g0348800*	id6008406	*GLK1*		73,152	4.4	regulation of plastid development, carotenoid production	Zheng et al., 2022; Li and Yao, 2022
*Os02g0182800*	id2002458	*HOS58*	*qTol2.1*	32,964	4.3	KNOX family class 2 homeodomain protein	
*Os02g0265200*	id2004522	*WRKY39*	*qAlph2.1*	48,328	4.2	heat responsive	Li et al., 2010

### Promoter and co-expression analysis

Promoter analysis of the tocopherol biosynthetic pathway genes (*vte1, vte2, vte3, vte4, vte5, vte6*, *hppd, tat, and ggpps*) was carried out using 1500 nucleotide (Wang et al., 2015) window upstream of the start codon of each gene to determine if corresponding *cis*-elements exists. The genes *ZFP15*, *ZFP182*, *NAC92*, *OsMADS59*, *HOS58*, and *WRKY39* have the binding site in the promoter region of different genes of the tocopherol biosynthesis pathway (**Supplementary Tables S5**, **S6**).

A co-expression analysis was performed for the different developmental stages of rice with a correlation threshold ≥ 0.6. The TFs identified in the GWAS analyses were checked via promoter analysis for common *cis*-elements. If they showed co-expression with tocopherol biosynthetic genes they were introduced as candidate TFs with probable roles in controlling tocopherol content. We identified 12 such TFs, including ERF, WRKY, TALE, bZIP, bHLH, NAC, MADS BOX, and MYB (**Table 2**).

### Epistatic interactions among QTLs

**Figure 4:**
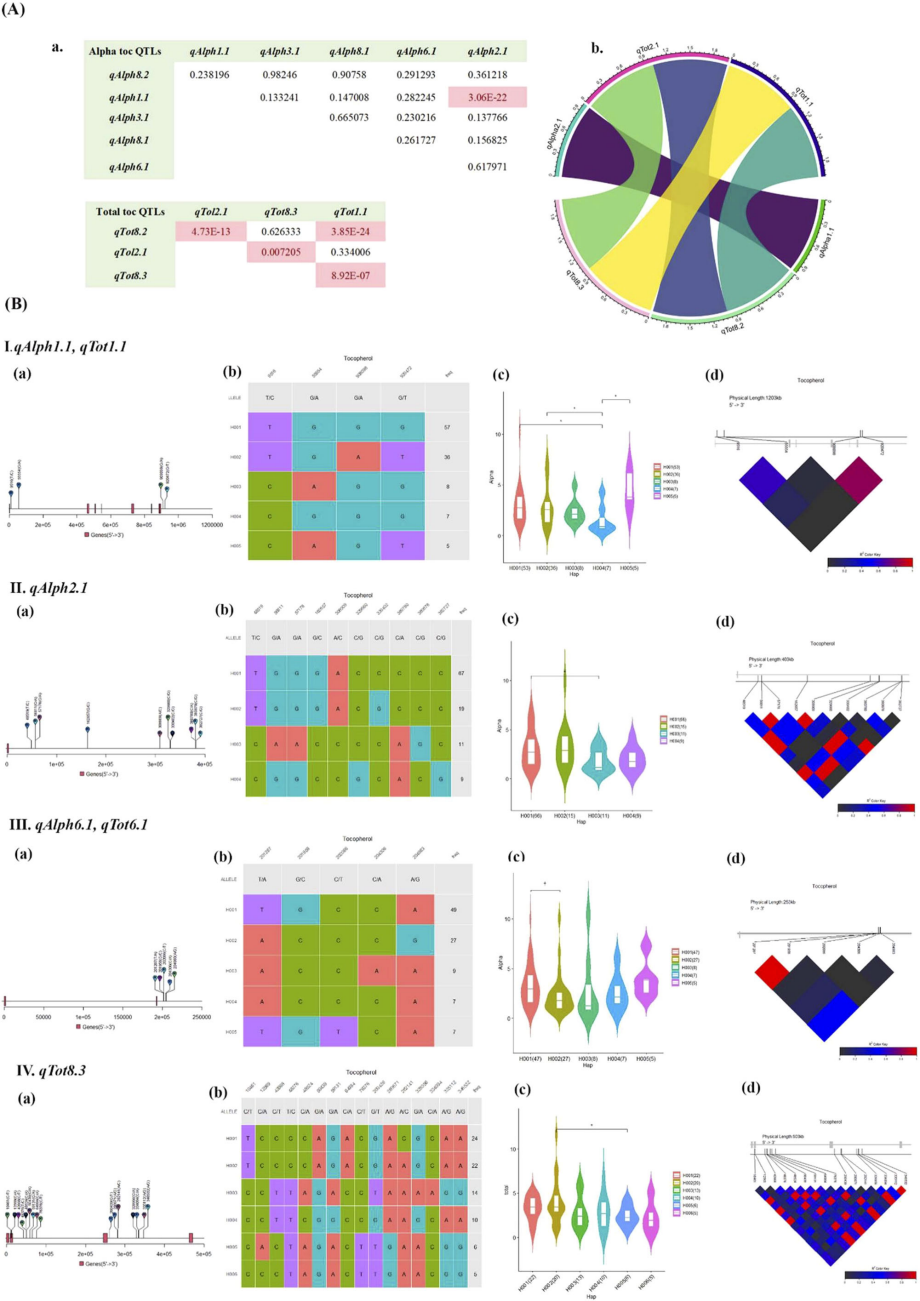
**(A)** a) Evidence of epistatic effects between alpha and total tocopherol QTLs. Interactions ≤ 0.05 are significant. (b) Circos plot of epistasis interaction between QTLs for alpha and total tocopherol detected from the GWAS result. QTLs that had significant epistatic associations are located around the circle. The Circlize package was used to visualize the significant epistatic interactions. **(B)** Haplotype analyses for different QTLs related to alpha and total tocopherols. (I) *qAlph1, qTot1.1*, (II) *qAlph2.1*, (III) *qAlph6.1, qTot6.1* and (IV) *qTot8.3.* (a) Visualization of SNP position above gene model, the black line represents the genome and rectangles represent exons; (b) Haplotype classification, each row represents a haplotype, colored columns represent loci, and the last column shows the frequency of each haplotype; (c) Phenotype comparisons among accessions possessing different haplotypes; (d) LD-block visualization of SNP sites in the locus. *Significant at *p* ≤ 0.05; † significant at *p* ≤ 0. 1. The red color indicates perfect LD, and the black color indicates no LD.

Complex traits in rice are regulated by epistatic interactions in addition to the additive effects of each gene, which makes the relationship between genotype and phenotype complex (Kato and Horibata, 2022). Here, six QTLs had epistatic effects (**Figure 4A**-a) between SNPs in QTLs *qAlph1.1* and *qAlph2.1* on chromosomes 1 and 2 affecting alpha-tocopherol (*p* ≤ 0.05), pairs of *qTot8.2* and *qTol2.1*; *qTot8.2* and *qTot1.1*; *qTol2.1* and *qTot8.3*; *qTot8.3* and *qTot1.1* on chromosomes 8, 1 and 2 all affecting total tocopherol (*p ≤* 0.05) (**Figure 4A**-b). The molecular basis behind these interactions may correspond with the regulation of gene expression and/or activity by *trans*-acting elements.

### Identification of favorable haplotypes of QTLs

To identify the cumulative effects of SNPs, haplotype analysis was conducted using all the QTLs although only statistically significant QTLs are being reported here. Haplotype analysis of QTLs *qAlph1.1* and *qTot1.1*, associated with alpha and total tocopherol on chromosome 1, formed a haplotype block with four SNP markers, which consisted of five haplogroups in our association panel (**Figures 4B**-I-a, b). All four markers within these QTLs showed substantial LD (**Figures 4B**-I-d), and variation in the haplotype alleles led to significant differences (*p* ≤ 0.05) between H001/H004, H002/H004, and H004/H005 haplotypes for both alpha and total tocopherol. The median of alpha and total tocopherol values in the violin plot of the haplogroups were 2.72 (H001), 2.65 (H002), 2.51 (H003), 1.34 (H004), 4.2 (H005) μg/g, respectively, and the highest amount of tocopherol was related to H005 (**Figures 4B**-I-c). H001 had the highest frequency (50.44%) and was mostly represented by *japonica* (TEJ) varieties based on the haplotype network (**Supplementary Figure S3**). Haplotype analysis of *qAlph2.1* resulted in four haplogroups with 10 SNPs in LD on chromosome 2 among the 179 rice accessions. The highest amount of tocopherol was associated with H002. The median of H001 and H003 were 2.65 and 1.32 μg/g, respectively, showing significant differences (*p* ≤ 0.05). The highest haplotype frequency was observed for H001 (63.0%), represented by TEJ, TRJ, and Admix varieties (**Figure 4B**-II-a-d, **Supplementary Figure S3**). Also, haplotype analysis of QTLs *qAlph6.1, and qTot6.1* associated with alpha and total tocopherol on chromosome 6, formed a haplotype block with five SNP markers, which consisted of five haplogroups in the association panel. All five markers were in LD, and variation in these haplotype alleles led to significant differences between the H001 and H002 haplotypes in terms of alpha and total tocopherol. The highest amount of tocopherol was found for the H005 haplotype, while the median values for H001 and H002 were 2.83 and 1.72 μg/g, respectively, showing a significant difference (*p* ≤ 0.1). H001 (32%), H002 (30%), and H003 (29%) have the highest frequency, which is represented by Indica, TRJ, and TEJ varieties, respectively (**Figure 4B**-III-a-d, **Supplementary Figure S3**). Haplotype analysis of *qTot8.3* resulted in the formation of six haplogroups based on 16 SNPs on chromosome 8. All 16 markers showed substantial LD. For total tocopherol, the median values among the H002 and H005 haplogroups were 3.51 and 2.40 μg/g, respectively, showing significant differences (*p* ≤ 0.05). The highest amount of tocopherol was related to H001. H001 (30%) and H002 (27%) have the highest frequency, which is represented by TEJ varieties (**Figure 4B**-IV-a-d, **Supplementary Figure S3**).

### Expression analysis of candidate genes

**Figure 5:**
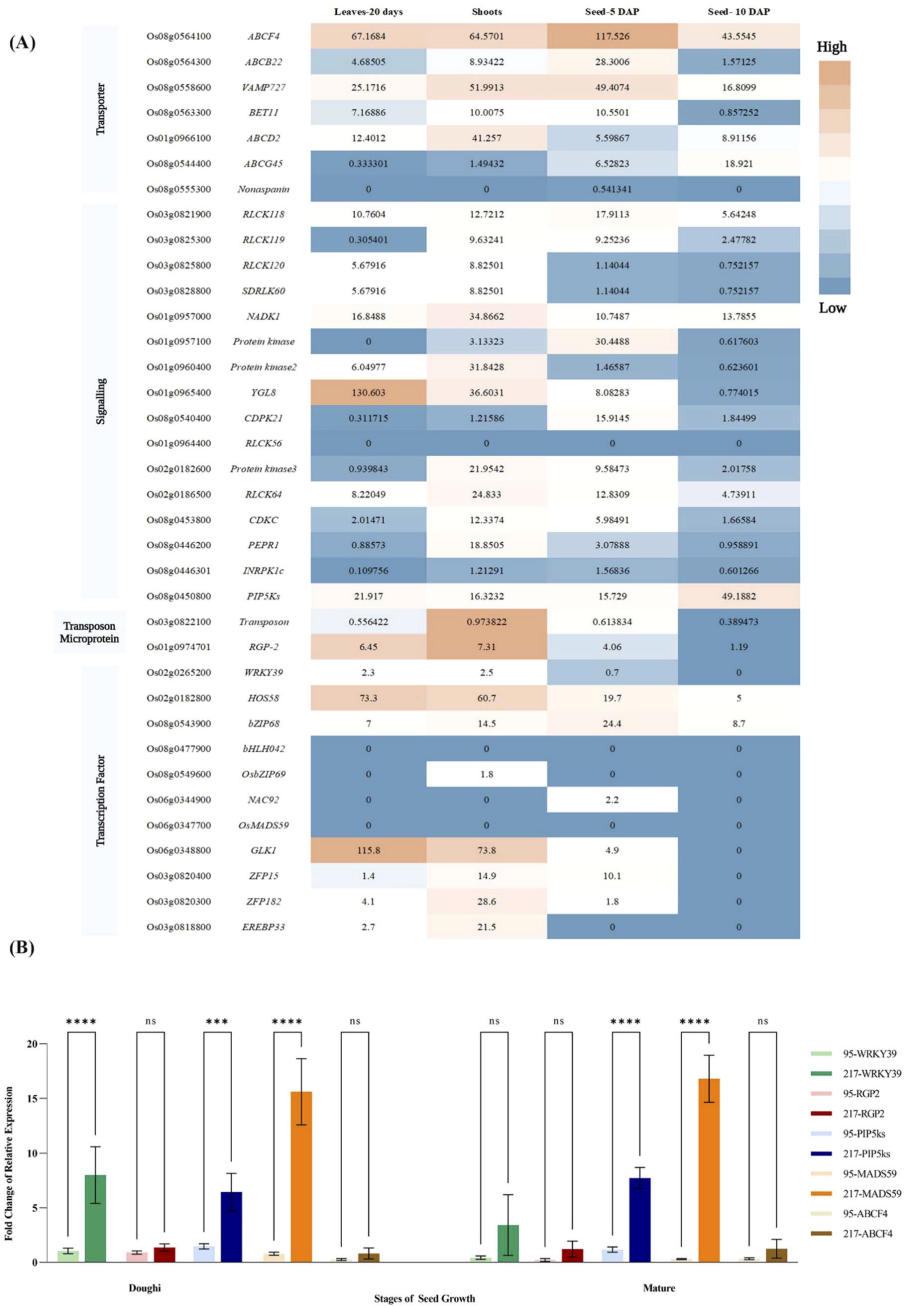
**(A)** Heatmap of candidate gene expression analysis by RNA-seq data obtained from RGAP database for seed 5 and 10 DPP, leaves (20-day-old), and shoots. Dark orange boxes indicate high transcript levels, and blue boxes indicate low transcript levels. **(B)** Expression analysis of *WRKY39*, *RGP*, *PIP5Ks*, *MADS59*, and *ABCF4* by real-time qRT-PCR. Amplification of cDNA from three developmental stages of seeds, *i.e*., milky, doughy, and maturity in two rice low tocopherol (g95) and high tocopherol (g217) content were carried out. A housekeeping gene, *OsActin*, was used as the control, and the expression data was compared to those of the milky stage. Data were analyzed using a two-way ANOVA, followed by Tukey’s multiple comparison test with a 95% confidence interval. Significantly different comparisons between #g95 vs #g217 at stages of seed growth are displayed on graphs using asterisks (***, *P* < 0.001; ****, *P* < 0.0001). No asterisk indicates that the difference is not significant. Error bars represent mean ± SD (*n* = 3 biological replicates).

To further verify the potential impact of the candidate genes on the regulation of tocopherol biosynthesis, the expression patterns of the candidate genes in various tissues [seeds (5 and 10 DPP), leaves (20-day-old) and shoots] based on data from the Rice Genome Annotation Project database were analyzed (**Figure 5A**). The results showed that among the transporter genes, *ABCF4* is expressed in all tissues, with the highest expression in the seed 5 DPP stage. *VAMP727* also showed a small extent of expression in all tissues, but the highest expression was in the shoot. *ABCG45* was slightly expressed in the seed 5 DPP and 10 DPP stages. The highest expression of *ABCD2* was in shoots, and *nonaspanin* was not expressed in any tissue we checked in this study. Among the signaling genes, *PIP5Ks* were expressed in all tissues; the highest expression was in seed 10 DPP. *RLCK118* was slightly expressed in all tissues. The highest expression in signaling genes belonged to *YGL8*, which was observed in leaves. Other genes that were involved in signaling were expressed in a small amount in different tissues. Transposon (*Os03g0822100*) and microprotein *RGP-2* also showed the highest expression in shoot tissue. Among TFs, the highest expression was found for *HOS58* in all tissues. *bZIP68* was weakly expressed in all tissues. The highest expression of *GLK1* was in leaves (20-day-old). The highest expression of *ZFP182* and *EREBP33* were observed in shoots. *ZFP15* was slightly expressed in shoots. Based on these results, two genes, *ABCF4*, and *PIP5Ks* from the transporter and signaling categories, respectively, were selected as candidates for validation using real-time qRT-PCR.

### Interaction network analysis

The STRING tool was employed to construct a protein association network between candidate genes and main genes (*VTE1*, *VTE4, HPPD*) involved in tocopherol biosynthesis. The results showed that *WRKY39*, *bHLH42*, *ZFP15*, and *ZFP182* are co-expressed with *VTE4*, and *ABCF4* and *RGP*-*2* are co-expressed with *VTE1*. Also, *RLCK119* is co-expressed with HPPD (**Supplementary Figure S4**). From these results, *WRKY39* and *RGP*-*2* genes in the transcription factor and microprotein categories were selected as the candidates for real-time qRT-PCR analysis.

### Predicting the effects of detected SNPs on gene function

Prediction of SNP effects was performed using SnpEff software. The predicted effects were categorized by impact as a modifier (with impact on noncoding regions), low (synonymous substitution), moderate (non-synonymous substitution), or high (disruptive impact on the protein). None of the associated SNPs fell within the sequence of the candidate genes, with two exceptions, namely dd1002410 and Id6008406, which were located in the intron region of *ABCD2* and *OsMADS59* genes, respectively. As a result, we selected *OsMADS59* for inclusion in the real-time qRT-PCR analyses.

### qRT-PCR validation of candidate genes

We used qRT-PCR to assess the relative expression levels of five candidate genes, including *WRKY39* (*Os02g0265200*), *RGP-2* (*Os01g0974701*), *PIP5K* (*Os08g0450800*), Os*MADS59* (*Os06g0347700*) and *ABCF4* (*Os08g0564100*) across different seed development stages (milky, doughy, maturity) in two accessions with high (g217) and low (g95) tocopherol contents, respectively (**Figure 5B**). The qRT-PCR results showed that the expression levels of all five selected genes were significantly higher in genotype g217 (high tocopherol) than g95 (low tocopherol) in both the doughy and mature stages. The highest expression was seen for *MADS59* in both stages. Two-way ANOVA analysis showed significant differences between the two genotypes g95 and g217 in the doughy stage for *WRKY39* (*P* < 0.0001), *PIP5Ks* (*P* < 0.001), and *OsMADS59* (*P* < 0.0001). Also, in the mature stage, two genes *PIP5Ks* and *OsMADS5*9 had statistically significant differences (*P* < 0.0001). *WRKY39* expression was not significantly different between the two genotypes in the mature stage. There was no significant difference between expression levels for the other genes. These results suggest that *WRKY39*, *PIP5Ks*, and Os*MADS59* may play a crucial role in tocopherol biosynthesis in rice seeds.

### Gene ontology enrichment and KEGG pathway determination

**Figure 6:**
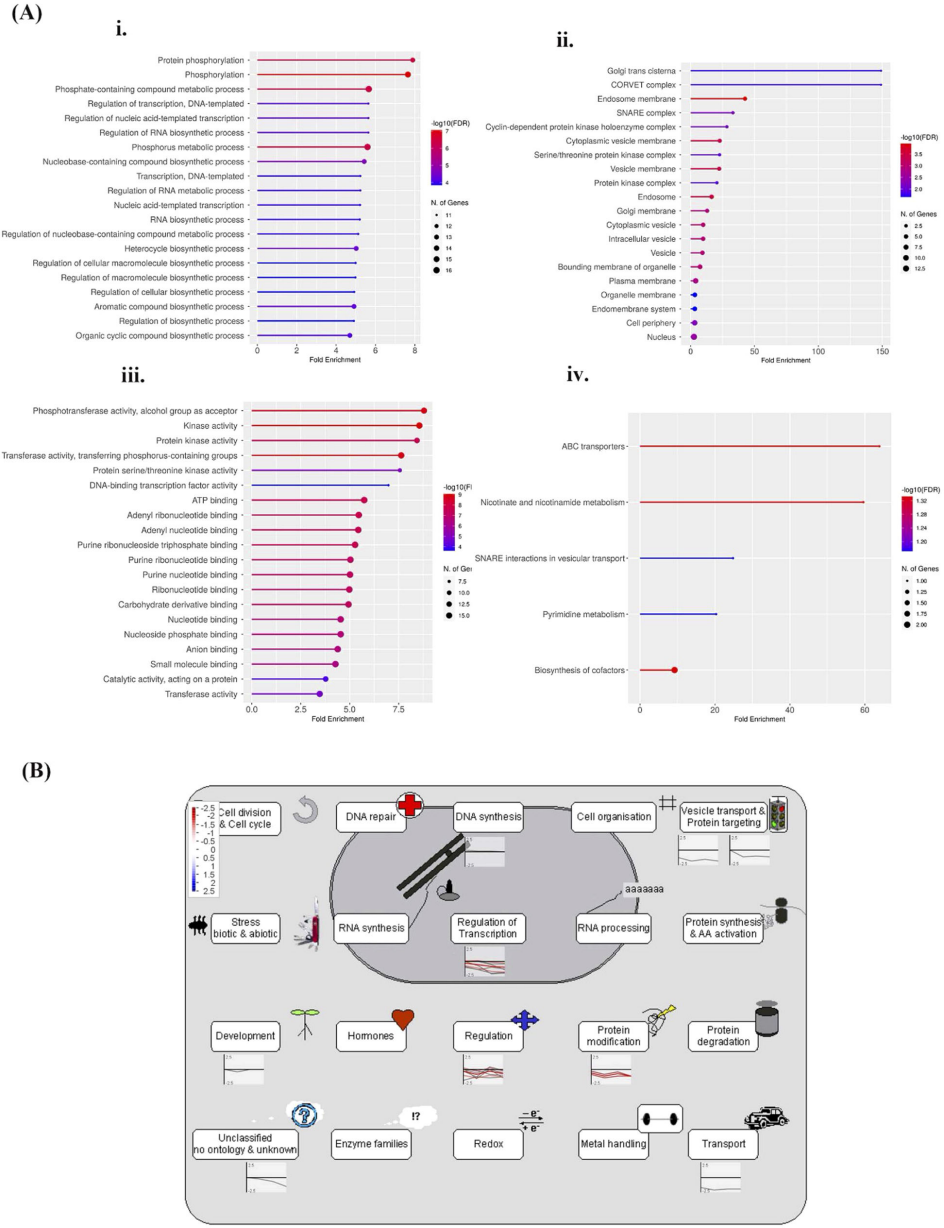
**(A)** Functional enrichment analysis of candidate genes given gene ontology (GO) and KEGG. GO analysis revealed that candidate genes were significantly enriched in (i) biological processes (ii) cellular components, (iii) molecular functions, and (iv) significantly enriched KEGG terms obtained from KEGG analysis. KEGG, Kyoto Encyclopedia of Genes and Genomes; GO, Gene Ontology. **(B)** MapMan analysis. Cell function overview associated with candidate gene. The studied genes are mainly effective in the regulation, protein modification, transport, and regulation of transcription, which are the precursors of tocopherol biosynthesis. For each functional group, the genes that increase and decrease more are shown as a series of blue and red lines. MapMan software (ver. 3.6.0RC1) was used to map transcriptomic data. Osa_RAPDB_mapping files were downloaded from the Map-Man store server (http://mapman.gabipd.org/).

To identify the pathways that had the most significant involvement of the identified genes that displayed differential expression (DEGs), candidate genes were submitted to ShinyGO v0.75 for GO and KEGG pathway analysis. GO analysis revealed that in the biological process category, the DEGs were enriched for involvement in protein phosphorylation, phosphorylation, regulation of transcription, phosphorus metabolic process, nucleobase-containing compound biosynthetic process, organic cyclic compound biosynthetic process, regulation of RNA biosynthetic process, nucleic acid-templated transcription and aromatic compound biosynthetic process (**Figure 6A**-i). The GO analysis showed that the candidate genes exhibited prominent enrichment in various CC terms for cellular components. These included endosome membrane, endosome, vesicle membrane, cytoplasmic vesicle, intracellular vesicle, bounding membrane of organelle, Golgi membrane, and plasma membrane (**Figure 6A**-ii). The molecular functions of candidate genes included kinase activity, phosphotransferase activity, ATP binding, adenyl nucleotide binding, purine ribonucleotide binding, protein serine/threonine kinase activity, transferase activity, DNA-binding transcription factor activity (**Figure 6A**-iii). KEGG pathway analysis demonstrated that candidate genes were significantly enriched in ABC transporters, biosynthesis of cofactors, nicotinate, and nicotinamide metabolism (**Figure 6A**-iv).

An overview of the candidate gene expression is provided in **Figure 6B**, which shows that they can be sorted into 9 BINs or subBINs, reflecting major cellular or functional processes. A high proportion of genes were involved in vesicle transport and protein targeting (*VAMP727*, *BET11*), regulation of transcription (*EREBP33*, *bHLH042*, *GLK1*, *HOS58*, *OsMADS59*, *WRKY39*, *bZIP68*), regulation (*PEPR1*, *RLCK56*, *RLCK120*, *PIP5Ks*, *CDPK21*), transport (*ABCF4*, *ABCB22*, *ABCD2*, *ABCG45*) and other functions, such as DNA synthesis and development. Only one gene (*ZFP182*) was included in BIN 35.1 (not assigned) and was not placed in any group. Marked changes in the expression pattern were found in all of these categories.

### GC-MS analysis

To study whether there is a relationship between the amount of vitamin E and the other fatty acids, one-step derivatization and GC-MS for the isolated grain oil of five selected rice genotypes (g57, g93, g170, g253, g275), were carried out. Rice seed fatty acid was mostly composed of palmitic acid (C_16:0_), oleic acid (C_18:1_), linoleic acid (C_18:2_), and stearic acid (C_18:0_) (**Table 3**). The latter was the least abundant (3.44-9.3%) in the five selected genotypes, while oleic acid was the most abundant (30.1-36.28%). According to our limited study, a positive correlation was observed between the amount of alpha and total tocopherol and C16:0, C18:1, and C18:2 fatty acids, but the correlation between the amount of tocopherol and C18:0 was negative. Overall, a positive correlation between total tocopherol and total fatty acid content was observed in rice oil in this study (**Supplementary Figure S5** **;** **Supplementary Table S7**).

**Table 3:** Fatty acid and tocopherol composition in rice oil (%) in selected accessions via GC-MS.

Tocopherol content (μg/g)			Main fatty acid in rice oil (%)			
Genotype	α-Toc	Total toc	Palmitic acid	Linoleic acid	Oleic acid	Stearic acid
g57	1.64	2.94	18.3	22.9	30.1	3.9
g93	0.076	0.23	17.17	28.99	36.28	3.8
g170	0.38	2.36	20.34	24.17	30.39	9.3
g253	30.83	31.76	18.14	27.78	33.75	3.44
g275	0.33	0.35	16.29	22.83	34.85	5.87

## Discussion

Tocopherols are important for grain nutrition quality and play an important functional role in human health due to their ability to prevent the oxidation of unsaturated fatty acids in cell membranes by scavenging free radicals (Shaw and Rajcan, 2017). For humans, daily tocopherol supplementation can decrease the risk of cancer and cardiovascular disease (Shaw et al., 2016). In plants, tocopherols protect chloroplasts from photooxidative damage during seed germination and improve oil stability in oilseeds (Morelli et al., 2023). Feedstock enriched with tocopherol improves and maintains growth and health in animals (Morand-Laffargue et al., 2023).

The tocopherol biosynthetic pathway, encoded by *VTE* genes (*VTE1-6*), is highly conserved in plants (Albert et al., 2022). The activity of *VTE7* in Arabidopsis and maize, an α/β hydrolase that interfaces with chlorophyll synthesis to release phytol, is unequivocal (Albert et al., 2022). Based on our alignment analysis (for both nucleotide and protein, data not shown) using in-house scripts in R, *VTE7* could not detected in the rice genome. This study found positive relationships between total fatty acids and total tocopherol content. According to a recent study (Chu et al., 2023) a positive correlation between total tocopherol and total fatty acids has also been observed in soja. Despite the availability of considerable information about vitamin E, our knowledge about the genetic architecture of vitamin E content in rice grains is rather rudimentary. This study aimed to employ GWAS to identify QTLs and candidate genes involved in the regulation, signaling, and transport of alpha and total tocopherols using natural rice accessions.

Association studies benefit from using genetically diverse germplasm, allowing for the examination of total allelic diversity derived from historical recombination events. In the present study, we implemented two different models, a univariate MLM model and a multivariate mixed model, FarmCPU. According to Segura et al. (2012), using the multivariate GWAS method addresses the issue of confounding between covariates and the test marker and lowers the false discovery rate (FDR) compared to univariate GWAS for the same significance threshold. This facilitates the detection of a greater number of QTLs. By examining Q-Q plots, the most suitable model for our data was FarmCPU. A panel of 179 rice genotypes was used to explore the diversity of tocopherol content in rice grain oil, using HPLC equipped with a fluorescent detector. Cluster analysis of the kinship matrix and PCA calculation indicated sufficient heterogeneity of genotypes that made it suitable for GWAS and allowed us to control for false associations due to the underlying genetic structure of the association population (Li et al., 2015).

Alpha and total tocopherol were detected similarly as in an earlier report by Lipka et al. (2015). The total tocopherol detected in this study ranged between 0.230 - 31.76 (µg/g). The dominant tocopherol isoform in rice seed oil (including the hull) was alpha-tocopherol (αT). Generally, tocopherol is higher in bran, but we couldn’t isolate the bran in this study. Therefore, our results may be slightly different from earlier studies (Wang et al., 2015). Quantification data clearly showed natural variations in the contents of tocopherols in our rice accessions (**Supplementary Figure S1**). Tocopherol content is a complex trait governed by several genetic loci. Phenotypic variation in the content and composition of tocopherols is primarily due to genetic effects, as shown by the high narrow-sense heritabilities (*H^2n^*) for alpha (0.79) and total (0.76) tocopherols. The values were not significantly different from bread-sense heritabilities (*H^2b^*), which suggests a minimal effect of other genetic factors, such as epistasis and dominance. High and stable heritability has always been useful for identifying strong associations between markers and traits of interest in association studies (Courtois et al., 2013). Significant SNPs (74) were identified using FarmCPU for alpha and total tocopherol content. In addition, unique QTLs (13) were detected, and the flanking sequences were searched for the genes associated with the putative function of defining alpha and total tocopherol content in rice grains. Another notable finding was that five pairs of epistatic QTLs were associated with tocopherol content. Epistasis is an important genetic component and a plausible feature of the genetic architecture of quantitative traits (Phillips, 2008). The change of a gene effect can often be mirrored in the placement and function of its signal-transduction pathway (Mackay et al., 2009). Here, the type of connection remains elusive. However, each QTL had several genes, and genes may have different epistatic effects. In the present study, 40 candidate genes were located within the confidence interval of the identified QTLs. Based on GWAS, 12 TFs were identified. Promoter analysis showed that *ZFP15*, *ZFP182*, *NAC92*, *OsMADS59*, *HOS58*, and *WRKY39* have binding sites in the promoter of genes involved in the biosynthesis of tocopherols. The members of the WRKY family of TFs, including *WRKY39* (*qAlph2.1*, ~48 kb away from SNP id2004522, associated with alpha-tocopherol), have been reported to participate in growth, development, metabolism, and responses to environmental cues (Phukan and Shukla, 2016; Chen et al., 2017), which again suggests the possible involvement of such TFs in regulating vitamin E content. *HOS58*, a TALE member, was identified as another candidate TF for regulating vitamin E content in rice grain. *HOS58* is located at *qTot2.1*, ~32 kb away from SNP id2002458, and was significantly associated with total tocopherol content. *ZFP15* and *ZFP182* of the C2H2 TF family were the other candidate TFs; both were common to two QTLs, including *qAlph3.1* and *qTot3.1* (~ 178 kb away from SNP id3016979). Members of this family in rice have been reported to be involved in response to abiotic stresses (Gourcilleau et al., 2011). *NAC92* (*qAlph6.1* and *qTot6.1*, ~ 201 kb away from SNP id6008406) and *OsMADS59* (*qAlph6.1* and *qTot6.1*, ~8 kb away from SNP id6008406) of MADS-Box family were the other TFs found in the close vicinity of the detected QTLs.

Haplotype analysis pinpointed the allelic variations with contributing factors in the tocopherol contents, which can be used for both pyramiding and marker-assisted breeding of rice (Varshney and Kumar, 2019). Four SNP combinations in the H005 haplogroup of *qAlph1.1* and *qTot1.1* QTLs had a greater effect than other haplotype groups. The combination of favorable alleles identified within the H002 haplotype was significantly higher for total tocopherol in *qTot8.3* QTL. Four SNP combinations within the H002 haplogroup of *qAlph2.1* QTL were more effective than other haplotype groups, and five SNP combinations within the H005 haplogroup of *qAlph6.1* and *qTot6.1* QTLs were more effective than other haplotype groups. These favorable alleles can be used in pyramiding into a target rice line by marker-assisted selection.

Studies in humans and animals have suggested that tocopherol-binding proteins (TBPs) are important for distributing and transporting alpha-tocopherol among different tissues (Niu et al., 2022). Bermúdez et al. (2018) identified the *SlTBP* (*Solanum lycoperisicum* tocopherol-binding protein) as a homolog of the human alpha-tocopherol transfer protein (*HsTTP*). *In vitro* biochemical assay suggested that *SlTBP* possesses alpha-tocopherol binding ability. *SlTBP* is chloroplast-targeted, and knocking down *SlTBP* expression in tomato conferred disorders in tocopherol, carotenoid, and lipid compositions (Bermúdez et al., 2018; Niu et al., 2022). Here, nine transporter genes from three families of ABC transporters, nonaspanins, and SNARE proteins on chromosomes 1 and 8 were identified as possible associations with tocopherol transport. The ATP-binding cassette (ABC) transporters are among the largest superfamilies involved in various biological processes (Akhtar and Turner, 2022). *ABCF4*, *ABCB22*, *ABCD2*, and *ABCG45* were found in our study (**Table 2**). Nonaspanins constitute a family of proteins known as TM9SF, which may function as a channel or small molecule transporter. Proteins in this group are endosomal integral membrane proteins (Sugasawa et al., 2001). *Os08g0554900*, *Os08g0555200*, and *Os08g0555300* belong to this family. Nonaspanin (*TM9SF*) and *VAMP727* co-located in QTLs *qAlph8.2* and *qTot8.2* with *~*88-119 kb and 252kb away from id8007643, respectively. SNARE protein (soluble *N*-ethylmaleimide-sensitive factor attachment protein receptor), is a highly conserved superfamily of proteins that mediate vesicle transport between endosomes and the trafficking to the plasma membrane of all eukaryotic cells (Jahn and Scheller, 2006). *VAMP727* and *BET11* were in this family. *BET11* is common in QTLs *qAlph8.2* and *qTot8.2*, ~165 kb away from id8007982.

Cellular signaling in plants is a complex phenomenon governed by several interconnected pathways. Protein kinases play pleiotropic roles in cells and act as predominant mediators in coordinating cellular responses to their environment. The kinase pathway is important for tocopherol biosynthesis, as the Arabidopsis *vte5* mutant contains reduced levels of tocopherol (Romer et al., 2024). In this study, *RLCK118*-*120* and *SDRLK60* were common in QTLs *qAlph3.1* and *qTot3.1*, ~65-95 and 210 kb away from id301697, respectively. *RLCK56* is located in QTLs *qAlph1.1* and *qTot1.1*, ~76 kb away from id1002410. *RLCK64* located in QTL *qTol2.1*, ~154 kb away from id2002458, was significantly associated with total tocopherol.

Receptor kinases (RKs) are of paramount importance in transmembrane signaling that governs plant reproduction, growth, development, and adaptation to diverse environmental conditions (Liang and Zhou, 2018). *NADK1* (*Os01g0957100*) and *CDKC1* (*Os01g0960400*) were common in two QTLs *qAlph1.1* and *qTot1.1*, ~31, 35, 80, 196 kb away from id1027324, respectively, that were significantly associated with alpha and total tocopherol. One of the key enzymes that regulate NAD(H)/NADP(H) balance is NAD kinase (NADK; EC 2.7.1.23), which catalyzes NAD phosphorylation in the presence of ATP (Takahara et al., 2010). Uridylate kinase is common in two QTLs *qAlph1.1* and *qTot1.1*, ~ 43 kb away from id1002410. The *UMP kinase* gene plays an important role in regulating chloroplast development and stress response in rice (Dong et al., 2019). *CDPK21* is located in QTL *qTot8.2*, ~150 kb away from id8007643, and was significantly associated with total tocopherol. Calcium-dependent protein kinases (CDPKs) play significant roles in regulating plant growth and development in response to various stresses, including drought (Mittal et al., 2017). CDKC located in QTL *qTot8.3*, ~141 kb away from id8006337 was significantly associated with total-tocopherol. Cyclin-dependent protein kinases (CDKs) form a conserved superfamily of eukaryotic serine/threonine protein kinases, which require binding to a regulatory cyclin for activity. CDKs are organized in several gene families and are involved in different aspects of cell biology, such as gene transcription, cell proliferation, and differentiation (Huang et al., 2008). *PEPR1*, *INRPK1c*, and *PIP5K* are located in QTL *qTot8.3*, ~42, 37, 206 kb away from id8006210, which were significantly associated with total tocopherol. Leucine-rich repeat receptor-like protein kinase (*PEPR1*) is a plant cell membrane-localized Leucine-rich repeat (LRR) receptor kinase that plays a critical role in plant innate immunity (Song et al., 1995). PIP5K or PI5K family regulates diverse cellular processes such as G protein-coupled receptor (GPCR) signaling, vesicle trafficking, chemotaxis, and cellular movement (Doughman et al., 2003; **Table 2**). MapMan is a useful tool to provide global views of diverse aspects of data and can be used to functionally classify rice genes (Usadel et al., 2009). Cell function analysis using the MapMan comprehensive overview highlighted the key pathways influenced by tocopherol biosynthesis.

qRT-PCR has been proposed for preliminary verification of candidate genes identified by GWAS (Li et al., 2024). In this study, we used qRT-PCR to preliminarily verify the association of five selected genes *WRKY39* (*Os02g0265200*), *RGP-2* (*Os01g0974701*), *PIP5Ks* (*Os08g0450800*), *MADS59* (*Os06g0347700*) and *ABCF4* (*Os08g0564100*) between the two accessions: g217 with high tocopherol and g95 with low tocopherol, on three different seed developmental stages (milky, doughy, maturity) of rice grain in the context of transcript analysis. Interestingly, we found that *WRKY39*, *PIP5Ks*, and *MADS59* displayed significant differences in the low and high tocopherol rice accessions.

The present study unveiled a rich source of genetic elements, including SNPs, QTLs, and putative candidate genes associated with tocopherol biosynthesis in rice. We validated three related tocopherol biosynthetic genes, including one signaling gene (*PIP5Ks*) and two TF genes (*WRKY39* and *MADS59*) that may be responsible for tocopherol variation in the natural rice population. In the future, the identified genes and QTLs can be used to develop molecular markers for marker-assisted selection (MAS), facilitating the selection of desirable traits in breeding programs. Additionally, multiple genes or QTLs controlling tocopherol content can be pyramided into a single genetic background to achieve synergistic effects and develop rice varieties with enhanced tocopherol levels. Advanced technologies such as CRISPR/Cas9 can also be employed to precisely edit the identified genes, creating targeted mutations to improve tocopherol content in rice. Last but not least, some of the identified genes can be implemented in the metabolic engineering of microorganisms for high-throughput production of tocopherols in an industrial manner.

## Conclusions

This study conducted GWAS analysis on a mapping population consisting of 179 rice accessions. We investigated regulatory genes (TFs, miPs, and transposons) and other genes functional in the transport and signaling of tocopherol biosynthesis in this natural rice population. Finally, three important candidate genes *WRKY39* (*Os02g0265200*), *PIP5Ks* (*Os08g0450800*), and *MADS59* (*Os06g0347700*), were identified and validated by qRT-PCR analysis. This study provides fundamental information for breeding new rice varieties with high tocopherol content and high-quality oils. Further functional studies, such as overexpression or knockout of these genes, will help us better understand the molecular mechanisms of tocopherol biosynthesis in rice.

## Data Availability

All relevant data can be found within the manuscript and its supporting materials and further inquiries can be directed to the corresponding author/s.
