# Supplementary material for: Genome-wide association study and genotypic variation for the major tocopherol content in rice grain
Source: Front Plant Sci. 2024 Oct 8;15:1426321. doi: 10.3389/fpls.2024.1426321 (PMC11493719; doi:10.3389/fpls.2024.1426321)
Supplement: Supplementary file 1 [file DataSheet1.docx]

# *Supplementary Figures*


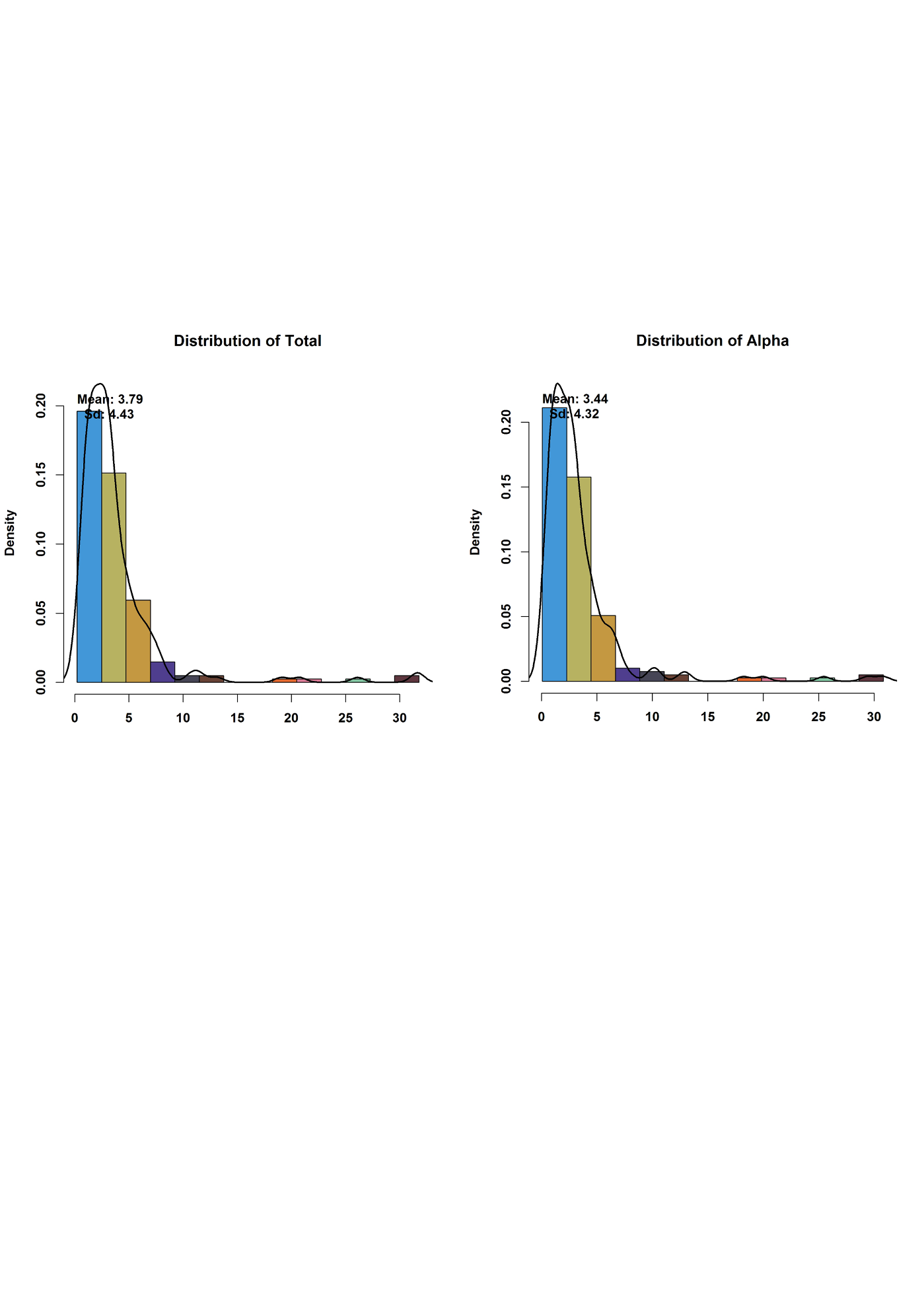


**Supplementary Fig. S1.** Phenotypic distribution of total and alpha-tocopherol.

**
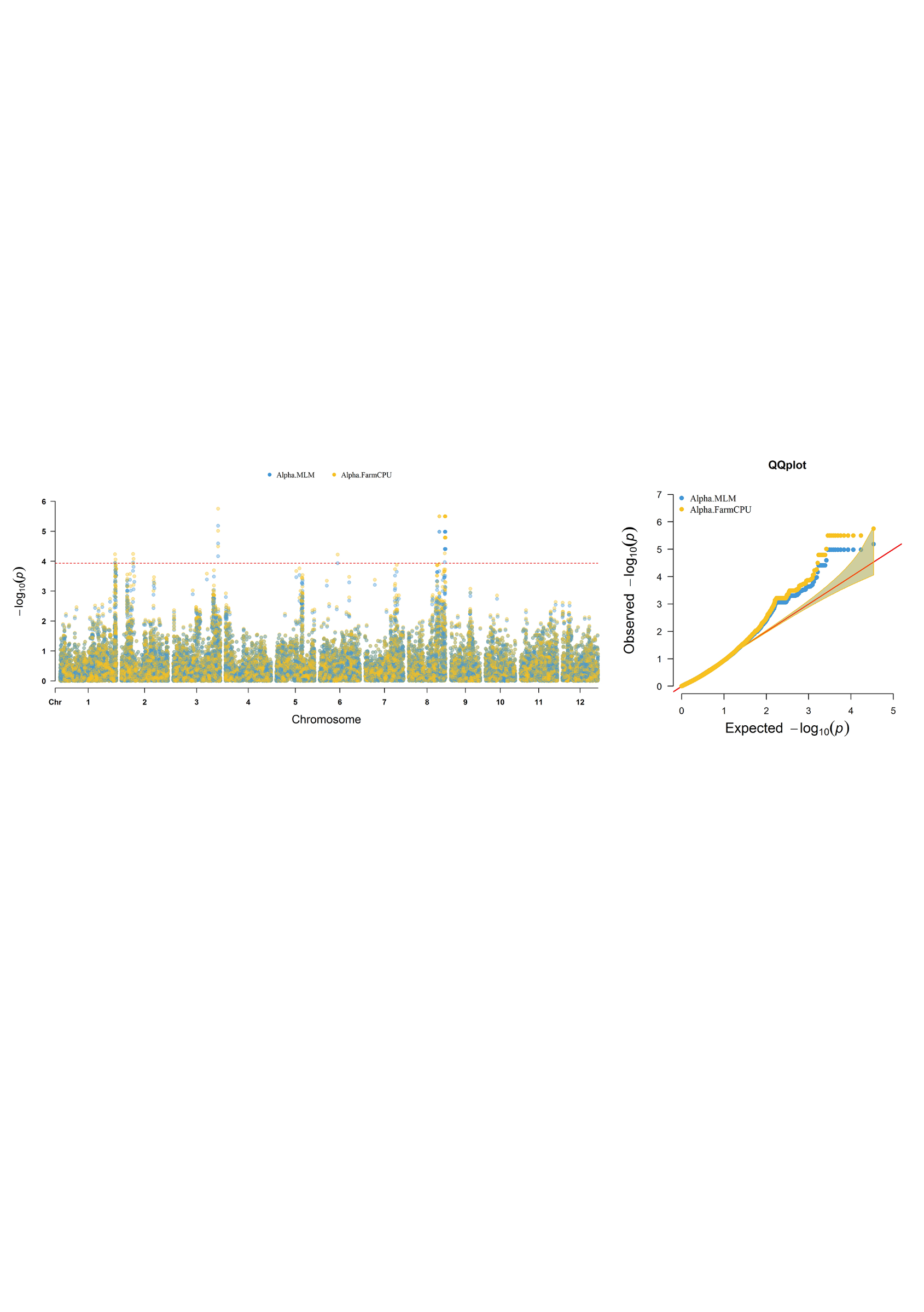
**

**Supplementary Fig. S2.** Comparing the Manhattan plots and Q-Q plots in MLM and FarmCPU models of Alpha-tocopherol

**
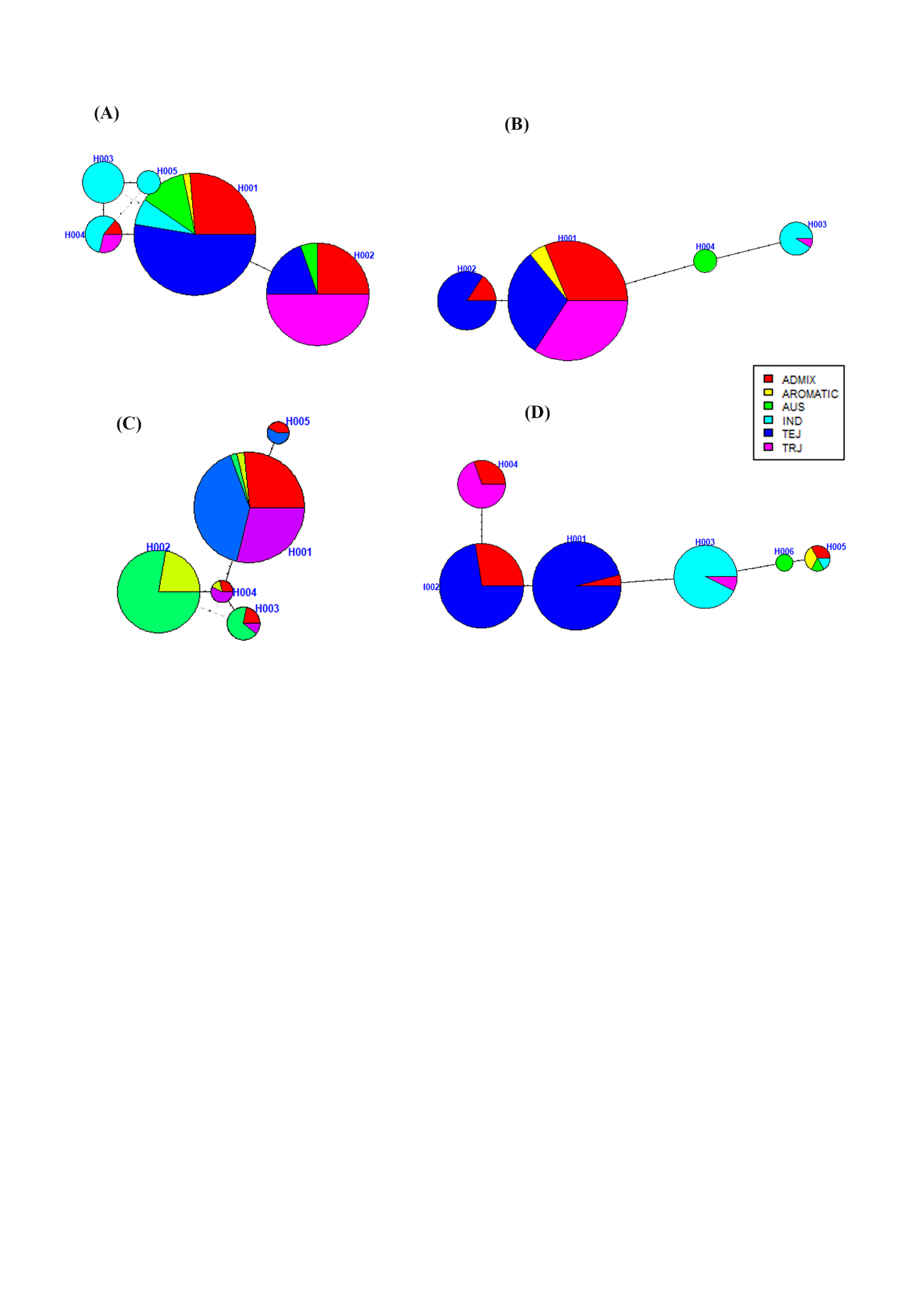
**

**Supplementary Fig. S3** haplotype network of **A)** ***qAlph1.1* and *qTot1.1* B) *qAlph2.1* C) *qAlph6.1, qTot6.1* D) *qTot8.3*** Each circle represents a haplotype and the size indicates accession number. The pies in different colors represent the ratio of category in each haplotype.


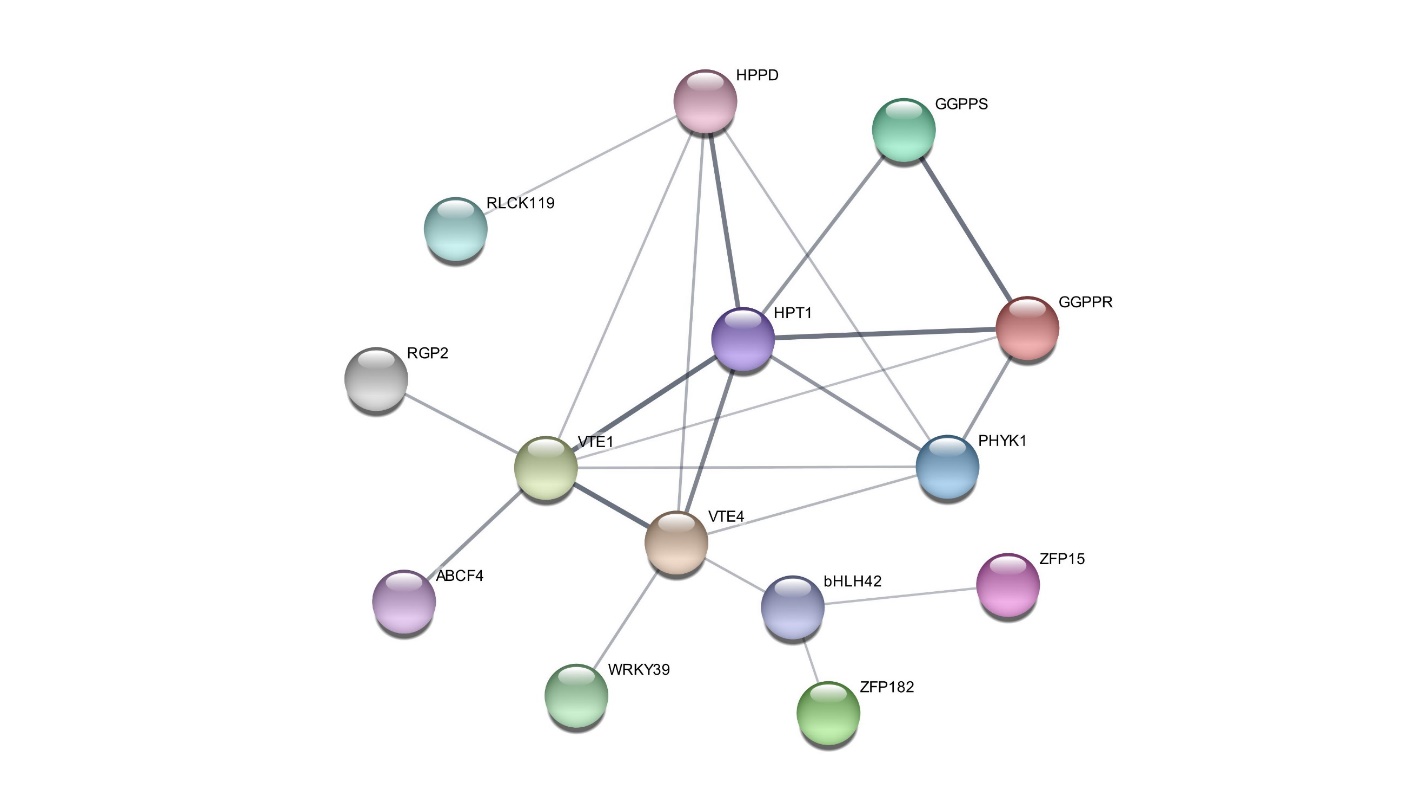


**Supplementary Fig. S4** Protein association networks between candidate genes and main genes (*VTE1*, *VTE4, HPPD*) involved in tocopherol biosynthesis. The results showed that *WRKY39*, *bHLH42*, *ZFP15,* and *ZFP182* are co-expressed with *VTE4,* and *ABCF4* and *RGP*-*2* are co-expressed with *VTE1*. Also, *RLCK119* is co-expressed with HPPD.


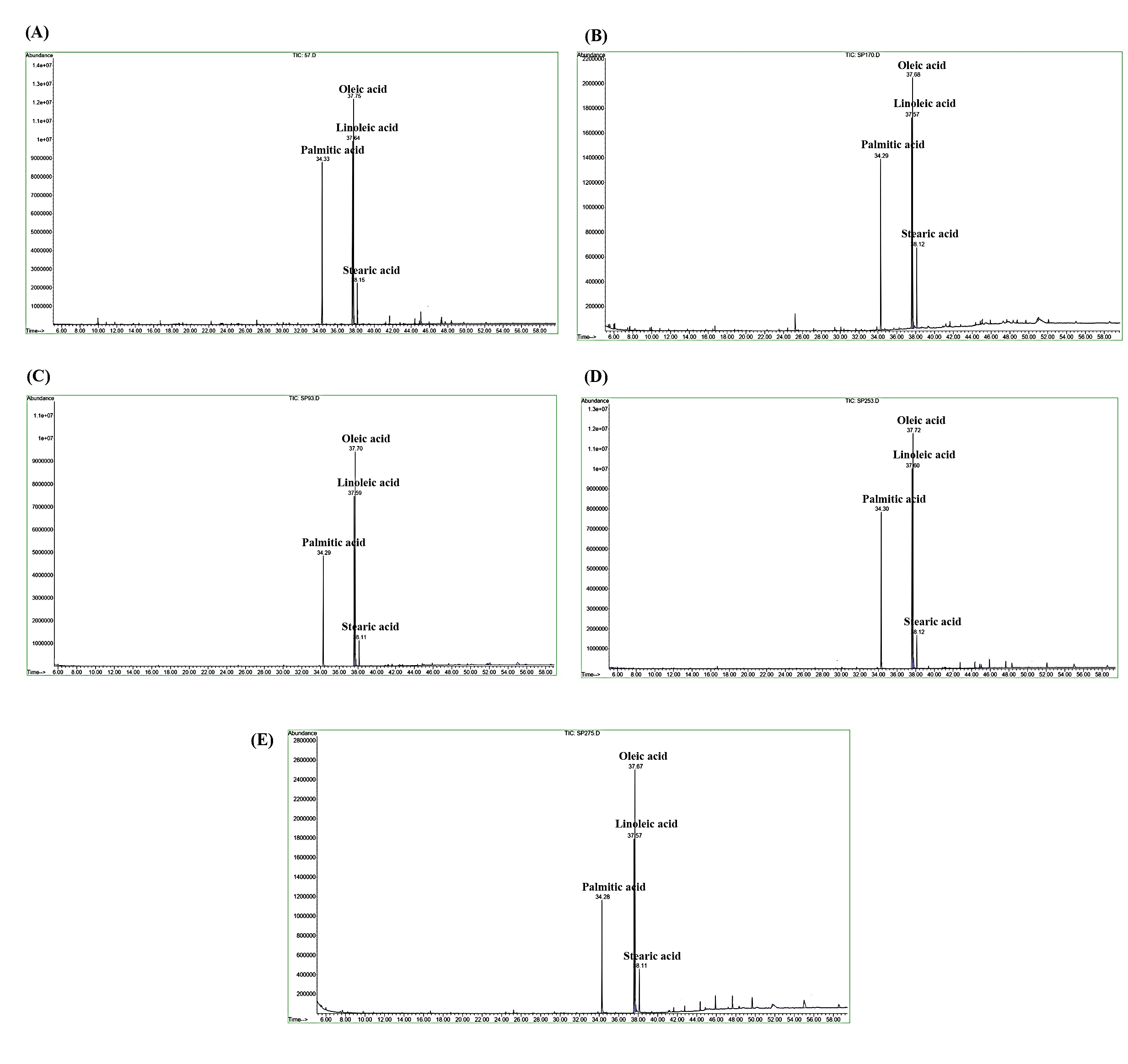
**Supplementary Fig. S5.** Comparison of GC-mass analysis between genotypes A: g57; B: g170; C: g93; D: g253; E: g275.

**linoleic acid**

**stc acid**
